# Differential genomic imprinting regulates paracrine and autocrine roles of IGF2 in mouse adult neurogenesis

**DOI:** 10.1038/ncomms9265

**Published:** 2015-09-15

**Authors:** S. R. Ferrón, E. J. Radford, A. Domingo-Muelas, I. Kleine, A. Ramme, D. Gray, I. Sandovici, M. Constancia, A. Ward, T. R. Menheniott, A. C. Ferguson-Smith

**Affiliations:** 1 Departamento de Biología Celular, Universidad de Valencia, Dr Moliner, 50, Burjassot 46100, Spain; 2 Department of Genetics, University of Cambridge, Downing Street, Cambridge CB2 3EH, UK; 3 Department of Obstetrics and Gynaecology, University of Cambridge, Robinson Way, Cambridge CB2 0SW, UK; 4 Centre for Trophoblast Research, University of Cambridge, Downing Street, Cambridge CB2 3EG, UK; 5 NIHR Cambridge Biomedical Research Centre, Hills Road, Cambridge CB2 0QQ, UK; 6 Department of Biology and Biochemistry, University of Bath, Claverton Down, Bath BA2 7AY, UK; 7 Murdoch Children’s Research Institute, Royal Children Hospital, Flemington Road, Parkville, Victoria 3052, Australia

## Abstract

Genomic imprinting is implicated in the control of gene dosage in neurogenic niches. Here we address the importance of *Igf2* imprinting for murine adult neurogenesis in the subventricular zone (SVZ) and in the subgranular zone (SGZ) of the hippocampus *in vivo*. In the SVZ, paracrine IGF2 is a cerebrospinal fluid and endothelial-derived neurogenic factor requiring biallelic expression, with mutants having reduced activation of the stem cell pool and impaired olfactory bulb neurogenesis. In contrast, *Igf2* is imprinted in the hippocampus acting as an autocrine factor expressed in neural stem cells (NSCs) solely from the paternal allele. Conditional mutagenesis of *Igf2* in blood vessels confirms that endothelial-derived IGF2 contributes to NSC maintenance in SVZ but not in the SGZ, and that this is regulated by the biallelic expression of IGF2 in the vascular compartment. Our findings indicate that a regulatory decision to imprint or not is a functionally important mechanism of transcriptional dosage control in adult neurogenesis.

## Abstract (web summary)

Selective biallelic expression of certain genes through genomic imprinting are known to play a role in controlling neurogenesis in the adult mammalian brain. Here the authors investigate the role of imprinting in the dosage control of Igf2 and its relevance for the function of IGF2 as a neurogenic regulator in the mouse brain.

## Graphical abstract

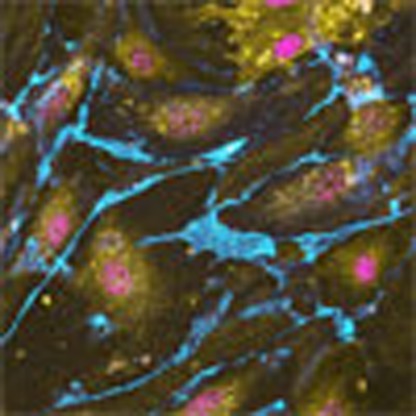

Stem cells are characterized by self-renewal and multipotency, and the stem cell niche is the functional microenvironment that enables their continuous self-renewal in response to physiological or pathological challenges^1^. Elucidating the components and regulation of the niche is important to understand disease processes and the development of future regenerative therapies. A number of factors regulate neural stem cell (NSC) function in these niches, including cytoarchitectural arrangement, extracellular matrix components, autocrine, paracrine and vascular factors^2^,^3^. The adult neurogenic niches consist of a complex collection of neural precursors, neuroblasts, glial cells and angioblasts in close proximity to small capillaries^4^,^5^. Neurogenesis occurs normally in the adult brain in two locations: the subventricular zone (SVZ) in the walls of the lateral ventricles (LVs)^6^ and the subgranular zone (SGZ) in the dentate gyrus (DG) of the hippocampus^7^. Neurons originating from the SVZ migrate to the olfactory bulb (OB) via the rostral migratory stream to generate new or replacement neurons^8^, whereas hippocampal neurogenesis is thought to play a role in learning and memory^9^,^10^. NSCs in the SVZ are glial fibrillary acidic protein (GFAP)-expressing radial glia-like cells^6^ that contact the LV apically, forming a pinwheel configuration surrounded by ependymal cells^11^. They extend long basal processes that contact blood vessels through specialized end-feet^5^. In the SGZ, NSCs also resemble astrocytes and extend a single radial process towards the molecular layer. A morphologically distinct class of progenitor cells that has horizontal processes has also been identified in the SGZ. The horizontal and radial progenitors have different proliferation rates and respond differently to neurogenic stimuli^12^, suggesting that in the SGZ there are different populations of progenitors with different properties. Dividing stem cells in the SGZ are also in close proximity to an extensive network of interconnected blood vessels and parenchymal astrocytes that can regulate their proliferation and differentiation via paracrine signalling^4^. This coupling of neurogenesis with angiogenesis, together with the close anatomical proximity of the SVZ and SGZ to blood vessels suggests a role for instructive vascular signalling in the regulation of NSC proliferation and/or differentiation^5^,^13^.

The ventricles contain cerebrospinal fluid (CSF) enriched with proteins secreted by the choroid plexus (CP) and brain vasculature. Many of these blood vessel and CSF-derived molecules are known to regulate progenitor cell proliferation during embryonic brain development, including fibroblast growth factors (FGFs), sonic hedgehog, bone morphogenic proteins, retinoic acid, Wnts and insulin-like growth factors (IGFs)^14^,^15^. Molecules in the CSF also influence adult neurogenesis in the SVZ as they bind to the primary cilia of adjacent SVZ progenitor cells, which extend into the ventricular space^14^,^16^. In contrast, neurogenesis in the DG may be less affected by CSF constituents. For example, intra-cerebroventricular infusion of exogenous epidermal growth factor and FGF2 expanded the SVZ precursor population, while no effect was seen in the more distant hippocampus^17^.

IGFs (IGF1 and IGF2) are mitogenic polypeptides with structural homology to pro-insulin; they signal predominantly via the IGF type-1 receptor (IGF1R) in the order of affinity IGF1>IGF2>insulin^18^. IGF2 also binds to the IGF type-2 receptor (IGF2R), which acts as a sink for excess IGF2 removal via internalization and lysosomal degradation^19^. IGF2 is well established as a critical factor regulating cell proliferation, growth, differentiation and survival. While fetal IGF2 is abundant, its concentration diminishes postnatally when growth-hormone-dependent IGF1 signalling dominates^20^,^21^. In humans, altered IGF2 dosage results in prenatal growth disorders and developmental defects^22^. However, in adults the expression of IGF2 is predominantly restricted to the brain where its function *in vivo* is poorly understood^20^,^23^. Lately, the hypothesis that the IGF system has a role in neurogenesis has gathered support^24^,^25^,^26^. IGF1, signalling through the IGF1R, is thought to influence embryonic brain growth and weight by reducing apoptosis and shortening the G1 cell-cycle stage in progenitor cells^27^,^28^. Furthermore, the CSF has been shown to have an age-dependent effect on embryonic cortical neural progenitor proliferation attributed to CP-secreted IGF2 (ref. 14). More recently, a functional role for IGF2 has been shown in memory^29^, where it is thought to act by increasing the survival of adult-born hippocampal neurons^30^. In addition, autocrine IGF2 seems to be crucial for maintaining NSC proliferation in the adult DG and promotes stemness of neural restricted precursors^24^,^31^. IGF2 has also been suggested to influence the adult brain under pathological conditions, as the uncontrolled proliferation that is characteristic of glioblastoma has been linked to elevated CSF–IGF2 concentration^32^. Together these studies implicate IGF2 as an important neurogenic regulator both during *in utero* development and in adult life.

Genomic imprinting is a process that causes genes to be expressed according to their parental origin, resulting in activation of one of the two alleles of a gene and repression of the other. In most human and mouse tissues, *Igf2* is expressed only from the paternally inherited chromosome^23^,^33^,^34^. However, it is specifically biallelically expressed (that is, from both the maternal and paternal chromosomes) in embryonic and postnatal human and mouse CP epithelium and leptomeninges^23^,^35^. Imprinting at the *Igf2* locus is mediated by the recruitment of the CCCTC-binding factor (CTCF) to the unmethylated maternal differentially methylated domain (DMD) restricting access of downstream enhancers to the *Igf2* gene promoters, thus preventing expression of the maternal allele^36^,^37^. However, biallelic expression of *Igf2* in the CP is regulated independently of the DMD, via a centrally conserved domain enhancer region^35^ located 5′ of the DMD and hence outside its influence. Nothing is known about the imprinting status of *Igf2* in the adult neurogenic niches.

We recently found that selective biallelic expression of another paternally expressed imprinted gene, *Dlk1*, in the SVZ is required for normal postnatal neurogenesis^38^, suggesting that selective brain-specific modulation of imprinting at other loci, such as *Igf2*, may also have a significant role in neurogenesis. Here, we consider the role of imprinting in the dosage control of *Igf2* and its relevance for the function of IGF2 as a neurogenic regulator. In this study, we demonstrate that the physiological absence of imprinting through the specific activation of the maternal *Igf2* allele in the brain vasculature and the CSF system, resulting in biallelic expression, is required for adult SVZ neurogenesis. In contrast, in the SGZ of the hippocampus where there is an autocrine requirement for lower levels of IGF2, its expression is monoallelic and in *Igf2* mutants, neurogenesis is perturbed only after deletion of the paternal allele. Our findings uncover the importance of genomic imprinting in the spatial and temporal dosage control of IGF2 acting through specific mechanisms to regulate the different neurogenic niches of the adult brain.

## Results

### Paternal and maternal expression of *Igf2* control brain growth

**Figure 1:**
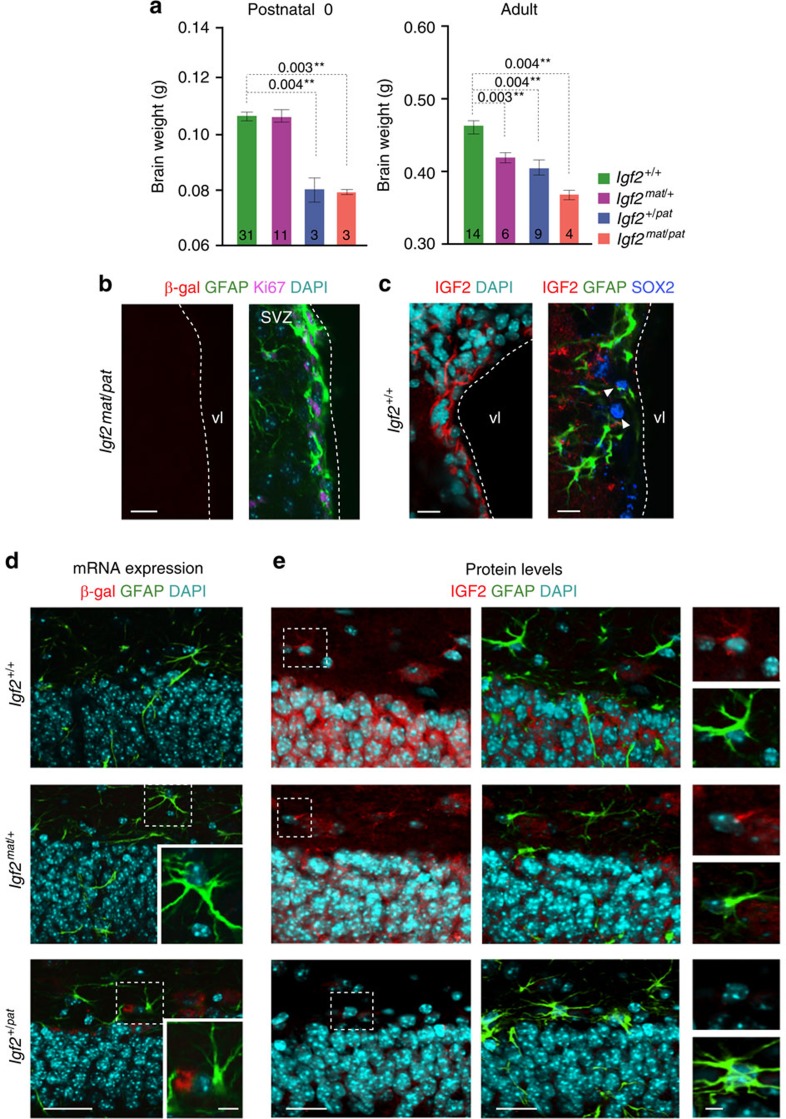
Paternal and maternal expression of *Igf2* differentially controls brain weights. (**a**) Brain weights in grams (g) of postnatal day 0 and adult (2 months old) wild-type (*Igf2*^+/+^), maternal (*Igf2*^*mat/+*^) and paternal (*Igf2*^*+/pat*^) heterozygous and homozygous knockout (*Igf2*^*mat/pat*^) mice. (**b**) Immunohistochemistry for β-galactosidase (red), Ki67 (pink) and GFAP (green) in the SVZ of homozygous knockout *Igf2*^*mat/pat*^ mice. (**c**) Immunohistochemistry for IGF2 within the SVZ of wild-type mice (left panel). Immunohistochemistry for IGF2 (red), GFAP (green) and SOX2 (blue) in the SVZ of wild-type mice. White arrowheads denote specific IGF2 staining in type-B NSC cells (right panel). (**d**) Immunohistochemistry for β-galactosidase (red) and GFAP (green) in the hippocampus of wild-type, maternal and paternal transmission heterozygote mice, showing expression only from the paternal allele. (**e**) Immunohistochemistry for IGF2 (red) and GFAP (green) within the hippocampus of *Igf2*^+/+^, *Igf2*^mat/+^ and *Igf2*^+/pat^ mice showing that *Igf2* is imprinted. DAPI was used to counterstain nuclei. vl, ventricle lumen. One-way analysis of variance and Tukey’s post-test. *P* values and number of animals per genotype or tissue samples are indicated. All error bars show s.e.m. Scale bars, 20 μm (**b**,**c**); 70 μm (**d**) (high-magnification images: 7 μm); 30 μm (**e**) (high-magnification images 7 μm). DAPI, 4,6-diamidino-2-phenylindole.

To characterize the functional role of *Igf2* imprinting in neurogenesis, we used a murine genetic model where the *Igf2* gene between exons 4 and 6 is replaced with a promoterless IRES:lacZneo cassette^39^. First, we confirmed the previously reported neonatal growth phenotypes upon paternal (*Igf2*^*+/pat*^) and maternal (*Igf2*^*mat/+*^) transmission of the mutation and in homozygous (*Igf2*^*mat/pat*^) mutant mice. As expected, body and brain weights were reduced in *Igf2*^*+/pat*^ mice at birth (postnatal day 0), while that of *Igf2*^*mat/+*^ mice was similar to their wild-type littermates, confirming that imprinted *Igf2* expressed from the paternal allele is important for prenatal somatic and cerebral growth (Fig. 1a; Supplementary Fig. 1). In contrast, in the adult, brain weights were reduced both in *Igf2*^*+/pat*^ and *Igf2*^*mat/+*^ animals, and further reduced in the *Igf2* homozygous mutant (Fig. 1a; Supplementary Fig. 1), indicating that biallelically expressed *Igf2* might control the growth of the developing brain specifically during the postnatal period.

We next assessed the cellular distribution and allelic expression of IGF2 in the adult brain, as this has not been described in detail. Analysis of *Igf2* expression by β-galactosidase staining in the forebrain of adult mice confirmed *LacZ* expression in the meninges, leptomeninges and CP epithelium in both *Igf2*^*+/pat*^ and *Igf2*^*mat/+*^, as previously described for the embryo (Supplementary Fig. 2a–e). To determine the cellular distribution of IGF2 expression at the protein level *in vivo*, immunostaining of IGF2 in combination with GFAP was performed on maternal and paternal heterozygote adult brains. IGF2 staining was observed in blood vessels of both *Igf2*^*+/pat*^ and *Igf2*^*mat/+*^ heterozygotes, revealing a previously undescribed biallelic expression of *Igf2* in the vasculature of both the developing and adult brain (Supplementary Fig. 3c). IGF2 expression from both parental chromosomes was also observed in meninges and CP (Supplementary Fig. 3a–c). This was confirmed by assessing *Igf2* imprinting status in tissues of wild-type F1-hybrid offspring from reciprocal crosses of *Mus musculus domesticus* (C57BL6/J) and *Mus musculus castaneus* (CAST/EiJ) strains (Supplementary Fig. 3d,e). Placental samples from reciprocal hybrids showed the expected paternally inherited imprinted expression of *Igf2*; however, meninges and CP from adult mice showed biallelic (non-imprinted) expression of the gene (Supplementary Fig. 3e).

The strength of this murine model is that combined analysis of β-galactosidase and IGF2 protein expression allows for the coincident delineation of IGF2 transcript and protein localization. We therefore analysed *Igf2* expression by performing β-galactosidase staining in *Igf2*^*mat/pat*^ mice, which showed no transcription of the gene in the SVZ (Fig. 1b); however, IGF2 protein could be readily detected by immunohistochemistry in the SVZ, both in the ependymal cells lining the ventricle and in GFAP+ type-B1 subependymal cells (Fig. 1c; Supplementary Fig. 4a). Importantly, in the SVZ we could detect cytoplasmic IGF2 in all of the GFAP+/Sox2+ cells in the absence of *Igf2*/β-gal expression (Fig. 1c), indicating cellular uptake of the protein from an exogenous source. In contrast, in the SGZ, β-galactosidase staining of adult maternal and paternal transmission heterozygous mice showed that endogenous *Igf2* is produced from the paternal allele in this niche (Supplementary Fig. 4b). These data were confirmed by *in vivo* immunostaining for the presence of β-galactosidase in combination with GFAP, demonstrating transcription of endogenous *Igf2* in the GFAP+ population in the hippocampus of the *Igf2*^*+/pat*^ mice (Fig. 1d). Consistently, IGF2 protein was also observed only from the paternal allele in the GFAP+ stem cell population of the DG (Fig. 1e; Supplementary Fig. 4c), indicating that in this niche canonically imprinted IGF2 functions in an autocrine manner.

### *Igf2* differential imprinting regulates SVZ and SGZ neurogenesis

**Figure 2:**
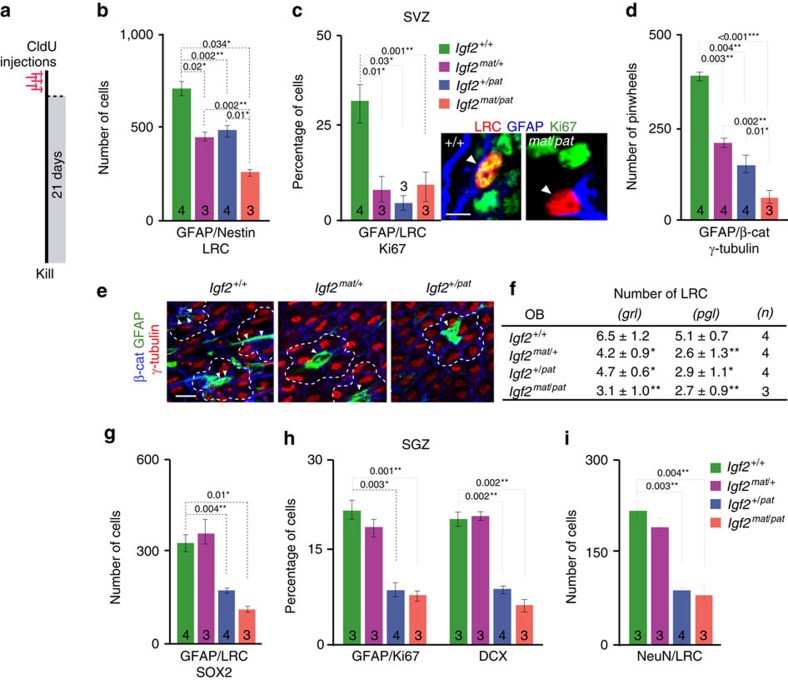
Biallelic and monoallelic expression of *Igf2* regulate SVZ versus SGZ adult neurogenesis. (**a**) Schematic drawing of the CldU injection protocol. (**b**) Quantification of the total number of CldU-label-retaining cells (LRCs) that are GFAP+/Nestin+ in the SVZ of *Igf2*^+/+^, *Igf2*^+/pat^, *Igf2*^mat/+^ and *Igf2*^mat/pat^ mice. (**c**) Quantification of the percentage of LRC/GFAP/Ki67+ cells in the SVZ of *Igf2* mutant mice (left panel). Fewer activated cells are observed in the absence of *Igf2*. High-magnification images of triple-positive LRC (red), GFAP (blue) and Ki67 (green) cells in wild-type and *Igf2*-deficient SVZ (right panel). (**d**) Quantification of the total number of GFAP+ NSCs contacting the ventricle in a pinwheel organization within the SVZ of the different *Igf2* mutant mice. (**e**) Whole-mount preparations of the SVZ wall immunostained for γ-tubulin (red), β-catenin (blue) and GFAP (green) in *Igf2* mutant mice. Arrowheads indicate the ventricle contacting γ-tubulin+ cilia of GFAP+ NSC cells (sagittal plane). (**f**) Quantification of the number of LRC reaching the granular (grl) or periglomerular (pgl) layers in the olfactory bulb (OB) of *Igf2*^+/+^, *Igf2*^mat/+^, *Igf2*^+/pat^ and *Igf2*^mat/pat^ mice. (**g**) Quantification of the total number of LRCs that are GFAP+/SOX2+ in the dentate gyrus of *Igf2*^+/+^, *Igf2*^mat/+^ and *Igf2*^+/pat^ heterozygotes and homozygous knockout *Igf2*^mat/pat^ mice. Maternal transmission of the mutation has no effect indicative of its imprinted repression on the maternal allele. (**h**) Quantification of the percentage of GFAP+ cells that also express Ki67 and the DCX+ cells in the hippocampus of the different *Igf2* mutant mice. (**i**) Quantification of the total number of newborn LRC–NeuN+ neurons in the SGZ of the hippocampus of *Igf2* mutant mice, showing a defect in the total neurogenesis only following paternal transmission of the mutation. 4,6-diamidino-2-phenylindole was used to counterstain nuclei. One-way analysis of variance and Tukey’s post-test. *P* values and number of animals analysed per genotype are indicated. All error bars show s.e.m. Scale bars, 7 μm (**c**); 10 μm (**e**).

To investigate the role of *Igf2* imprinting in the neurogenic niches *in vivo*, we analysed the SVZ and SGZ of wild-type *Igf2*^*+/pat*^ or *Igf2*^*mat/+*^ mice^39^. *Igf2* homozygous null animals were also analysed. Two-month-old mice were injected with the nucleotide analogue CldU 3 weeks before killing (Fig. 2a). In the SVZ, fast-proliferating transit-amplifying progenitors dilute out the CldU, which is only retained in slowly proliferating NSCs (label-retaining cells, LRCs) and OB newborn neurons that ceased to divide and terminally differentiated soon after the injection^40^. *Igf2* heterozygous mice showed a specific reduction in the proportion of GFAP+/SOX2+ and GFAP+/Nestin CldU-LRCs that was less proliferative as measured by the cell-cycle antigen Ki67 (Fig. 2b,c; Supplementary Fig. 5a–c), suggesting that IGF2 regulates the cycling of activated B cells. Consistent with this, a reduced number of type-B1 NSC γ-tubulin+ apical contacts in the anterior medial wall of the LV^11^ were evident in both maternal and paternal *Igf2* heterozygous mice (Fig. 2d,e). Importantly, *Igf2* homozygous mutants showed a more severe decrease in the number of CldU-LRCs, indicating a dosage-dependent mitogenic effect of *Igf2* on NSC proliferation (Fig. 2b–d). As a consequence of the reduced number of NSCs in the *Igf2* mutant SVZs, we found a reduction in the percentage of MASH1+ transient amplifying progenitors and of the DCX+ neuroblast population (Supplementary Fig. 5d). This resulted in fewer newborn neurons reaching the OB, visualized by a highly significant decrease in the numbers of postmitotic CldU+ newly formed neurons in the granular and periglomerular layers of the mutant OBs (Fig. 2f; Supplementary Fig. 5e). In agreement with this, the number of primary neurospheres obtained *ex vivo* was also reduced, regardless of the parental origin of the mutation (Supplementary Fig. 5f,g), supporting a role for IGF2 derived from both parental chromosomes in regulating the number and cycling of activated NSCs within the SVZ.

In the DG of the SGZ, two major classes of cells were expected to retain CldU labelling: slowly dividing GFAP/SOX2+ NSCs that do not dilute the CldU through divisions (LRCs) and newborn neurons that incorporated CldU in the granular layer before cell-cycle exit. Quantification of the LRCs in the DG showed that the number of GFAP+/Sox2+/LRC cells in the SGZ was significantly reduced in the paternal heterozygous and homozygous knockout *Igf2* mice (Fig. 2g), combined with a specific proliferation defect in GFAP+ cells as measured by Ki67 labelling (Fig. 2h). Strikingly, no differences in the maternal heterozygous compared with wild-type SGZ were found (Fig. 2g,h), consistent with *Igf2* imprinting in the SGZ. As a consequence of the fewer NSCs in the paternal heterozygotes and homozygous mutant mice, we found a reduction in the percentage of the DCX+ neuroblast population (Fig. 2h) resulting in reduced neurogenesis as visualized by a significant decrease in the number of postmitotic NeuN+/LRC newly formed neurons in the granular layer of the mutant hippocampus (Fig. 2i). Supporting the *in vivo* data, the number of *ex vivo* primary neurospheres obtained from the SGZ was reduced in the paternal heterozygotes and homozygous mutant mice, whereas no changes were found in the maternal heterozygotes (Supplementary Fig. 5f,g). This parental-origin-dependent decrease in the number of stem cells and their proliferation rate in the SGZ in *Igf2* mutant mice indicate that, in contrast to the SVZ, canonically imprinted, paternally expressed autocrine *Igf2* plays an important function in regulating adult hippocampal neurogenesis *in vivo*.

### Paracrine and autocrine IGF2 regulate SVZ- and SGZ-derived NSCs

**Figure 3:**
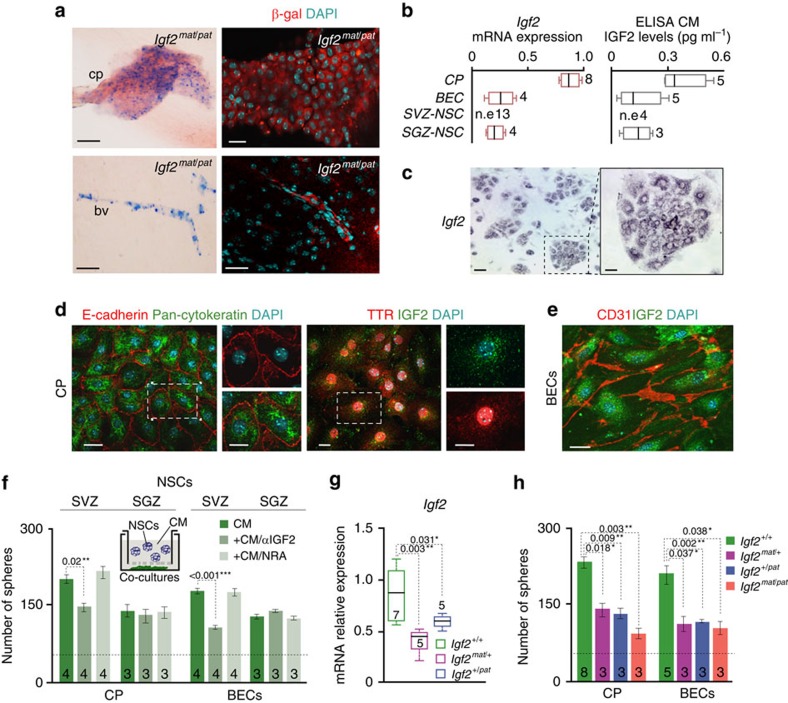
NSCs derived from SVZ but not from the SGZ respond to endothelial IGF2. (**a**) β-galactosidase staining (blue) in the dissected adult choroid plexus (CP) and brain vasculature (bv) of *Igf2*^mat/pat^ mice (left panels). Immunohistochemistry for β-galactosidase (red) within the adult CP and bv in homozygous knockout *Igf2*^mat/pat^ mice confirms expression of the gene (right panels). (**b**) Quantitative PCR (qPCR) of *Igf2* expression in brain endothelial cells (BECs) and epithelial cells from adult CP and NSCs derived from the SVZ and hippocampus of adult wild-type mice (left panel). Data are expressed relative to *Gapdh*. IGF2 protein detection by ELISA in conditioned media (CM) produced from NSCs, BECs and epithelial cells from adult CP (right panel). (**c**) *In situ* hybridization for *Igf2* in 3-week-old primary CP cultures. (**d**) Immunocytochemistry for E-cadherin (red) and pan-cytokeratin (green) labelling epithelial cells from CP primary cultures (left panel). Immunocytochemistry for IGF2 (green) and transthyterin (TTR; red) showing a high-magnification view of transthyretin-positive epithelial cells expressing IGF2 (right panel). (**e**) Immunocytochemistry for IGF2 (green) and CD31 (red) in BECs. (**f**) Quantification of the number of neurospheres derived from the SVZ or the SGZ and formed in co-cultures with CP or BECs. Anti-CREB antibody was used as a non-related antibody (NRA) negative control. Inset shows schematic drawing of the co-culture experimental set-up. (**g**) qPCR of *Igf2* expression in CP isolated from wild-type, *Igf2*^+/pat^, *Igf2*^mat/+^ and *Igf2*^mat/pat^ mice, normalized to β-actin. (**h**) Quantification of the number of neurospheres formed in co-cultures with CP epithelial cells or BECs derived from *Igf2* mutant mice. Dashed line indicates number of spheres formed in non-co-culture conditions. DAPI was used to counterstain nuclei. One-way analysis of variance and Tukey’s post-test. *P* values and number of animals per genotype or tissue samples are indicated. All error bars show s.e.m. Scale bars, 100 μm, upper left panel; 50 μm, upper right and lower left panels; 10 μm, lower right panel (**a**); 60 μm (high-magnification image: 30 μm) (**c**); 30 μm (high-magnification images: 15 μm) (**d**); 20 μm (**e**). DAPI, 4,6-diamidino-2-phenylindole.

On the basis of IGF2 *in vivo* localization, we hypothesized that IGF2 is biallelically produced from the neurogenic niche promoting self-renewal and proliferation of SVZ NSCs in a paracrine manner and monoallelically expressed in the hippocampus of SGZ NSCs in an autocrine manner. NSCs located in specific neurogenic niches proliferate and self-renew in close apposition to capillaries, and some endothelial-derived molecules have been shown to regulate NSC behaviour^4^,^5^,^13^,^16^. Indeed, our data indicate that although adult SVZ NSCs do not produce endogenous IGF2, they may take up the factor from the blood vessels and/or the CSF, two IGF2-expressing compartments in direct contact with the adult stem cells (Fig. 3a). However, NSCs from the SGZ that produce endogenous IGF2 may not be equally influenced by niche-secreted IGF2. To further test this hypothesis, we isolated NSCs from wild-type adult SVZ and SGZ and grew them *in vitro* as floating neurospheres^40^. In agreement with the *in vivo* expression data, SVZ neurosphere cultures did not express *Igf2* (Fig. 3b). However, as previously described^14^,^31^, addition of recombinant IGF2 protein to insulin-free neurosphere cultures resulted in 70% more neurospheres that were markedly bigger and incorporated more bromodeoxyuridine (BrdU), effects that were not due to a survival effect as no changes in the number of apoptotic or necrotic cells were observed after treatment (Supplementary Fig. 6a–c). In contrast, neurospheres isolated from the SGZ expressed endogenous IGF2 and did not respond to the addition of recombinant IGF2 protein to the cultures (Supplementary Fig. 6c,d). Furthermore, we detected IGF2 by enzyme-linked immunosorbent assay (ELISA) in conditioned media of primary neurospheres acutely isolated from adult SGZ but detected none in the conditioned medium of SVZ primary neurospheres, supporting the hypothesis that NSCs from the SGZ produce endogenous IGF2 (Fig. 3b).

To determine the capacity of NSCs to respond to endothelial-produced IGF2 *in vivo*, we co-cultured wild-type adult NSCs with primary CP epithelial cells (CP) or brain endothelial cells (BECs) acutely isolated from 1-month-old wild-type mice (Fig. 3c–f)^41^,^42^. These co-cultures induced a significant increase in SVZ neurosphere formation that was partially abrogated by the addition of IGF2-blocking antibody (Fig. 3f). No effects were observed in NSCs derived from the SGZ (Fig. 3f). Consistent with this, similar co-culture experiments of wild-type NSCs with *Igf2* mutant-derived CP epithelial cells or BECs, known to express reduced levels of *Igf2* (Fig. 3g), showed a reduced response in SVZ neurosphere formation compared with wild-type co-cultures (Fig. 3h), supporting the dose-sensitive role of endothelial-produced IGF2 in SVZ-NSCs self-renewal. These data together suggest that IGF2 acts as a niche-secreted NSC paracrine regulator and that the absence of *Igf2* imprinting is necessary in the SVZ throughout the life course, whereas no paracrine effects for IGF2 were observed in the SGZ neurospheres.

**Figure 4:**
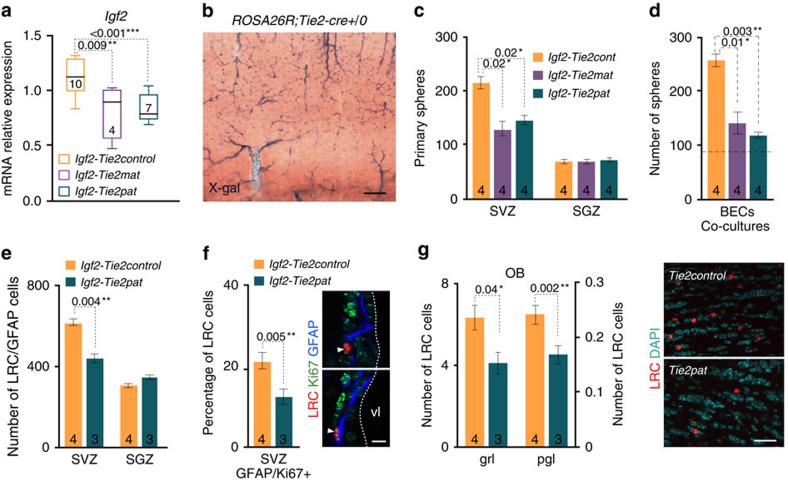
IGF2 secreted by the endothelial niche regulates NSC self-renewal specifically in the SVZ. (**a**) *Igf2* expression in control (*Igf2-Tie2control*), floxed maternal allele (*Igf2-Tie2mat*) and floxed paternal allele (*Igf2-Tie2pat*) mice, normalized to *Pecam1*, expressed specifically in blood vessels. (**b**) X-gal histochemistry (blue precipitate) in the adult cortex (Cx) of ROSA26R;Tie2-cre +/0 mice showing the specific expression of the *Tie2-cre* transgene in blood vessels. (**c**) Quantification of the number of primary neurospheres generated from the SVZ and the SGZ of the different *Igf2* conditional mutant mice. (**d**) Quantification of the number of neurospheres formed in co-cultures with BECs derived from *Igf2* conditional mutant mice. Dashed line indicates number of spheres formed in non-co-culture conditions. (**e**) Quantification of the total number of LRC in the SVZ and SGZ of *Igf2-Tie2control* and *Igf2-Tie2pat* mice. (**f**) Quantification of the percentage of LRC/GFAP cells that are Ki67+ in the SVZ of *Igf2-Tie2control* and *Igf2-Tie2pat* mice (left panel). Immunohistochemistry for LRC (red), GFAP (blue) and Ki67 (green). Arrowheads show some examples of LRC/GFAP+ cells that are not actively cycling in the *Igf2-Tie2pat* SVZ (right panel). Fewer activated cells are observed when IGF2 production from blood vessels is decreased. (**g**) Quantification of the number of LRC reaching the granular (grl) or periglomerular (pgl) layers in the OB of *Igf2-Tie2control* and *Igf2-Tie2pat* mice (left panel). Immunohistochemistry showing BrdU-LRC (red) in the OB granular layer of *Igf2-Tie2control* and *Igf2-Tie2pat* mice (right panel). DAPI was used to counterstain nuclei. One-way analysis of variance and Tukey’s post-test. *P* values and number of animals per genotype or tissue samples are indicated. All error bars show s.e.m. Scale bars, 50 μm (**b**); 15 μm (**f**); 30 μm (**g**). DAPI, 4,6-diamidino-2-phenylindole.

To further test whether brain endothelial IGF2, a previously undescribed source of IGF2, regulates NSC behaviour *in vivo*, we performed a conditional mutagenesis experiment in which we crossed mice carrying loxP sites flanking the *Igf2* gene on the maternal (*Igf2*^*flox/+*^) or paternal (*Igf2*^*+/flox*^) allele^43^ with mice expressing Cre-recombinase under the control of the endothelium-specific angiopoietin receptor tyrosine kinase receptor-2 (Tie2/Tek) promoter (*Tie2-cre*^*+/0*^)^44^. This resulted in the reduction of IGF2 expression (both RNA and protein) in CD31+ endothelial cells of blood vessels in the striatal side of *Igf2*^*flox/+*;^*Tie2-cre*^*+/0*^ (referred to as *Igf2-Tie2mat*) and *Igf2*^*+/flox*;^*Tie2-cre*^*+/0*^ (referred to as *Igf2-Tie2pat*) compared with *Igf2*^*flox/flox*^*;Tie2-cre*^*0/0*^ (*Igf2-Tie2control*) mice (Fig. 4a). We confirmed that the floxed allele had effectively recombined in endothelia by performing X-gal histochemistry in the adult brain of ROSA26R;*Tie2-cre+/0* mice (Fig. 4b). We next examined the role of endothelial IGF2 in the regulation of NSC maintenance and proliferation in the two neurogenic niches. The number of primary neurospheres obtained *ex vivo* from the SVZ of *Igf2-Tie2mat* and *Igf2-Tie2pat* animals was reduced, whereas no changes in the hippocampal neurosphere number were found (Fig. 4c). To directly test whether these effects on SVZ neurogenesis were due to a reduction in the paracrine IGF2 produced and secreted by blood vessels within the niche, we grew monolayers of BECs from the *Igf2-Tie2mat* and *Igf2-Tie2pat* mice and co-cultured them with wild-type neurospheres. As described before, co-cultures with wild-type BECs increased the neurosphere formation rate. However, this increase was not observed in co-cultures with either Igf2*-Tie2mat* or *Igf2-Tie2pat* BECs (Fig. 4d). Finally, 2-month-old mice were injected with the nucleotide analogue BrdU 3 weeks before killing. We quantified the total number of BrdU-LRC/GFAP-positive cells and found a reduction in the SVZ, whereas no changes were observed in the adult hippocampus *in vivo* (Fig. 4e). This reduction in the SVZ stem cell population was associated with reduced proliferation of the NSC pool, as fewer GFAP/LRC that were Ki67+ were found (Fig. 4f). As a result, the number of postmitotic BrdU+ newly formed neurons in the granular and periglomerular cell layers of the *Igf2-Tie2pat* OB was decreased (Fig. 4g). No changes in the number of new neurons incorporated into the SGZ were observed (Supplementary Fig. 7). This confirms that endothelial-derived IGF2 regulates NSC maintenance in SVZ but not SGZ neurogenesis, and that this is regulated by the biallelic expression of IGF2 in the vascular compartment.

### Monoallelic and biallelic *Igf2* act via IGF1R activation

**Figure 5:**
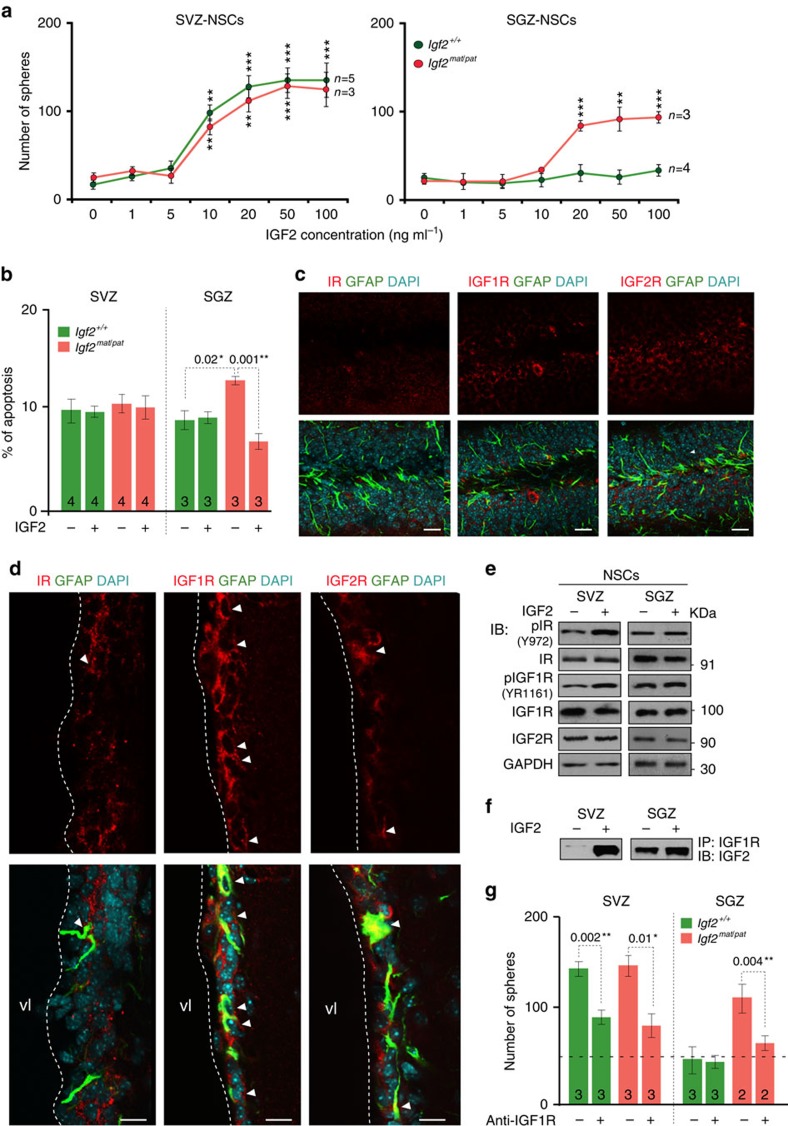
Monoallelic and biallelic expression of *Igf2* involve different mechanisms of action. (**a**) Quantification of the number of neurospheres formed after treatment with increasing concentrations of exogenous IGF2 of NSCs derived from wild-type *Igf2*^*+/+*^ and *Igf2*^*mat/pat*^ SVZ and SGZ. All treatments were done in the absence of insulin and in combination with EGF and FGF in the media. (**b**) Percentage of apoptotic bodies in 2-day *in vitro Igf2*^*+/+*^ and *Igf2*^*mat/pat*^ SVZ and SGZ neurospheres treated or not with 50 ng ml^−1^ of recombinant IGF2. (**c**) Immunohistochemistry for IR, IGF1R or IGF2R (red) and GFAP (green) in coronal sections of the adult wild-type SGZ and SVZ (**d**). Arrowheads indicate receptor expression in NSCs. (**e**) Immunoblot of phospho-IR (pIR), phospho-IGF1R (pIGF1R) and IGF2R in both SVZ and hippocampus-derived neurospheres in the presence or absence of IGF2 (left panel). (**f**) Immunoblot (IB) for IGF2 after the immunoprecipitation (IP) with IGF1R antibody in wild-type SVZ and SGZ-derived NSCs cultured in the absence or presence of exogenous IGF2. (**g**) Quantification of the number of neurospheres formed in response to IGF2 treatment in the absence or presence of a blocking antibody for IGF1R, both in SVZ and SGZ-NSCs cultures derived from *Igf2*^*+/+*^ and *Igf2*^*mat/pat*^ mice. DAPI was used to counterstain nuclei. vl, ventricle lumen. One-way analysis of variance and Tukey’s post-test. *P* values and number of cultures per genotype are indicated. All error bars show s.e.m. Scale bars, 20 μm (**c**,**d**). DAPI, 4,6-diamidino-2-phenylindole.

We sought to characterize the downstream pathways responding to the differential regulation of *Igf2* expression in the two neurogenic niches. Consistent with a paracrine and not autocrine role for IGF2 in the SVZ, neurospheres isolated from homozygous *Igf2* mutant SVZ displayed a similar self-renewal capacity to wild type upon culture with exogenous IGF2. However, in the absence of endogenous expression of *Igf2*, mutant SGZ NSCs responded to treatment with recombinant IGF2 (Fig. 5a), perhaps suggesting that the unresponsiveness observed in the hippocampus-derived NSCs might be caused by a saturation of the IGF2 signalling pathways in the wild-type cultures. IGF2 can also function as a survival factor; hence, to determine the potential role of IGF2 in NSC survival, we quantified the number of apoptotic bodies in the wild-type and *Igf2* mutant neurosphere cultures in the presence or absence of recombinant IGF2. Notably, no changes were observed in SVZ NSCs, but an increase in the percentage of apoptosis was observed in SGZ-NSCs *Igf2*^*mat/pat*^ cultures. This effect was rescued by addition of exogenous IGF2 (Fig. 5b), supporting a different role for IGF2 in the two neurogenic niches: a mitogenic paracrine in the SVZ and an autocrine role as a survival factor in the SGZ.

The IGF system consists of two ligands and multiple signalling receptors with different downstream effectors. More specifically, IGF2 binds with high affinity to IR-A, IGF1R and to the hybrid receptor and can interact with IGF2R to target the ligand to lysosomes for degradation^18^,^19^. At the messenger RNA level, IR-A, IR-B, IGF1R and IGF2R have similar expression in the SVZ and SGZ niches, with IR-A and IGF1R being the most strongly expressed receptors (Supplementary Fig. 8a). Consistent with this, GFAP+ cells in the SVZ and SGZ expressed both IR and IGF1R proteins (Fig. 5c,d). Treatment of SVZ-derived neurospheres with 20 ng ml^−1^ of recombinant IGF2 increased phospho-IR and phospho-IGF1R levels, whereas no effect was observed on SGZ-derived neurospheres (Fig. 5e; Supplementary Fig. 8b). No changes on IGF2R were observed in either case. These data suggest that paracrine IGF2 is taken up by the SVZ NSCs via activation of IR and/or IGF1R. To further study the potential function of IGF1R in NSC biology, we performed an immunoprecipitation using an IGF1R antibody followed by immunoblotting for the presence of the ligand in NSCs from SVZ and SGZ. Neurospheres were cultured in the absence or presence of recombinant IGF2. In the absence of IGF2, SVZ NSCs do not show binding of IGF2 to the IGF1R, whereas binding to the receptor was clearly observed after IGF2 treatment (Fig. 5f). Importantly, in SGZ NSCs the ligand appears bound to the IGF1R before IGF2 treatment, indicating an interaction between endogenous IGF2 and the receptor and suggesting that ligand-binding sites on the receptor are already occupied by autocrine IGF2 (Fig. 5f). Hence, despite the demonstrable presence of the IGF1R in wild-type SGZ-derived neurospheres, exogenous IGF2 has no effect on either neurosphere growth or intracellular signalling, consistent with saturation of the signalling pathways by autocrine production of IGF2 in the wild-type SGZ. To determine whether the IGF1R was required for the effects of IGF2 on neurosphere formation, we performed a receptor inhibition experiment using IGF1R antibodies in the neurosphere formation assay. In the SVZ NSCs, treatment with IGF2 promoted neurosphere formation and blocking IGF1R binding impaired this in both wild-type and *Igf2* mutants. The promotion of SGZ sphere formation by exogenous IGF2 in *Igf2*-deficient NSCs was also mediated by IGF1R binding (Fig. 5g). This suggests that in both neurogenic niches, IGF2 acts primarily via the IGF1R to promote postnatal neurogenesis.

It has previously been shown that IGF2 acts via AKT to regulate NSC proliferation^24^. To determine whether this molecular mechanism underlies the differential action of IGF2 on NSCs within the two niches, we analysed the role of AKT-dependent signalling after acute knockdown of *Igf2* in neurospheres derived both from the SVZ and the SGZ with a lentivirus expressing a silencing short hairpin RNA (shRNA) directed against *Igf2* messenger RNA (Supplementary Fig. 8c). Although no changes in phospho-AKT (pAKT) were observed after *Igf2* knockdown in SVZ-derived neurospheres, addition of exogenous IGF2 did increase pAKT levels in these cells (Supplementary Fig. 8d), supporting the paracrine role of IGF2 in the SVZ NSCs. *shRNA*-mediated knockdown of *Igf2* also led to a reduction in pAKT levels in hippocampal NSCs (Supplementary Fig. 8d), supporting the survival effect of IGF2 in the SGZ NSCs. In conclusion, IGF2 functions in a paracrine and autocrine manner in the SVZ and SGZ, respectively, mediated by specific activation of IGF1R and subsequent induction of the pAkt downstream pathway.

## Discussion

This study shows that biallelic versus monoallelic expression of *Igf2* in the adult brain is functionally and specifically important *in vivo*. We demonstrate that the physiological temporal and region-specific modulation of *Igf2* imprinting plays a key role in regulating NSCs in the adult SVZ and SGZ neurogenic niches. In the majority of tissues, particularly during fetal development, *Igf2* expression occurs solely from the paternal allele^23^,^33^,^34^. The key findings of our study are that functionally important *Igf2* is not only expressed from the paternally inherited allele but also from the maternally inherited allele in the vasculature, CP, meninges and leptomeninges of the adult brain, and such biallelic expression is required to support SVZ neurogenesis. *In vitro* explant models have shown that IGF2 from the embryonic CSF influences the proliferation of cortical progenitors^14^. Our results significantly extend these findings, showing that *Igf2* deficiency *in vivo* impairs the activation of the adult stem cell pool in the SVZ under physiological conditions, most likely due to the lower concentration of IGF2 secreted by the vasculature and the CP. Using the conditional deletion of *Igf2* from the brain vascular compartment, we demonstrate the previously unrecognized functional importance of the endothelium compartment as a source of paracrine IGF2 in SVZ neurogenesis. In contrast, we show that SGZ NSCs rely on canonically imprinted autocrine IGF2 expressed only from the paternal allele.

Previous studies showed that shRNA-mediated knockdown of *Igf2* specifically in adult mouse DG and SVZ led to reduced NSC proliferation in the DG, but had no effect on SVZ-NSCs^24^. Our findings explain this observation and illustrate the sophisticated regulation of IGF2 dosage through imprinting modulation in the two neurogenic compartments. We propose a model in which monoallelic *Igf2* expression is sufficient to maintain IGF2 concentration at the site of action in autocrine SGZ signalling. In contrast, we suggest that paracrine signalling via the CSF and endothelial compartment requires biallelic *Igf2* expression to maintain a sufficiently high concentration of IGF2 at the target NSCs following diffusion within the SVZ (Supplementary Fig. 10). We demonstrate that IGF2 acts via the IGF1R in both neurogenic niches, but that in the wild-type SGZ this signalling pathway is saturated by autocrine IGF2, rendering it unresponsive to exogenous or vascular-derived IGF2 both *in vivo* and *in vitro*. In addition, we show that IGF2 acts as a self-renewal promoting molecule in the SVZ, whereas in the SGZ the autocrine IGF2 prevents NSCs from apoptosis.

The role of genomic imprinting in gene dosage control is not fully understood. It is interesting to consider whether biallelic expression evolved from the imprinted state, or represents preservation of an ancestral mode of regulation maintained because the mitogen demand in the SVZ renders the evolutionary cost of functional haploidy too high in these tissues. It is possible that imprinted expression in the SGZ represents an evolved mechanism to limit IGF2 dosage as a means to prevent overstimulation of the autocrine signalling axis. Regulation of adult neurogenesis through the selective absence of IGF2 imprinting is reminiscent of recent work showing that selective biallelic expression of another imprinted gene, *Dlk1*, is required for adult neurogenesis^38^. Together these findings suggest that genomic imprinting can be used as a mechanism of gene dosage control in particular developmental contexts and raises questions about the evolution, adaptability and flexibility of imprinting as an epigenetically regulated process that can respond to intrinsic environmental cues. Further studies will determine whether non-cerebral stem cell niches can also modulate genomic imprinting, and will investigate the signalling processes and epigenetic mechanisms underlying imprinting dynamics.

## Methods

### Animals and *in vivo* manipulations

The generation of *Igf2* mutant mice has been described previously^39^. Mice were maintained on a C57BL6 background and crossed with CD1 to generate viable adult offspring of wild-type (*Igf2*^+/+^), maternal (*Igf2*^mat/+^) and paternal (*Igf2*^+/pat^) transmission heterozygotes and homozygous null *Igf2*^*mat/pat*^ mutant mice. *Igf2* mutant animals were genotyped by PCR analysis of DNA, extracted from mouse ear-punch tissue with the following primers: *Igf2-E6F* (5′-TGGCCTGGTATCCAAAACAT-3′), *Igf2-E6R* (5′-CTGGATGACATGGACAGTGG-3′) and Neo-E6F (5′-AGCGCATCGCCTTCTATC-3′). *Tie2-cre*^*+/0*^*(B6.Cg-Tg (Tek-cre)1Ywa/J)* mice were obtained from The Jackson Laboratory. *Igf2*;*Tie2-cre floxed* mice were generated as described previously^43^, and genotypes were determined with the following primers: for the deletion, *II-delF* (5′-TTACAGTTCAAAGCCACCACG-3′), *II-delRW* (5′-GCCAAAGAGATGAGAAGCACC-3′) and *II-delRD* (5′-GCCAAACACAGTAAAAAGAAATGC-3′). For the transgene, oIMR1084F (5′-GCGGTCTGGCAGTAAAAACTATC-3′) and oIMR1085R (5′-GTGAAACAGCATTGCTGTCACTT-3′). Internal control, oIMR7338F (5′-CTAGGCCACAGAATTGAAAGATCT-3′) and oIMR7339R (5′-GTAGGTGGAAATTCTAGCATCATCC-3′). See also ‘Imprinting assay’ below. Housing of mice and all experiments were carried out in accordance with UK and Spanish Government Home Office licensing procedures.

### Immunostaining and β-galactosidase histochemistry

Thymidine analogues administration regimes have been previously detailed^38^,^40^. Briefly: mice at 2–4 months of age were injected intraperitoneally with 50 mg of 5-chloro-2′-deoxyuridine (Sigma) or BrdU (Sigma) per kg of body weight every 2 h for 12 consecutive hours (7 injections in total). At 30 days after the injections, animals were deeply anaesthetized and transcardially perfused with 4% paraformaldehyde (PFA) in 0.1 M phosphate buffer pH 7.4 (PB) and brains were vibratome-sectioned at 40 μm. For immunohistochemistry, sections were washed in PBS and blocked at room temperature for 1 h in PBS (0.9% NaCl in PB) with 0.1% Triton X-100 supplemented with 10% FBS and then incubated overnight at 4 **°**C with primary antibodies (Supplementary Table 1). For CldU or BrdU detection, sections were pre-incubated in 2 N HCl for 30 min at 37 °C and neutralized in 0.1 M sodium borate (pH 8.5). For BrdU incorporation analysis *in vitro*, neurospheres cultured for 48 h in complete medium were treated with 2 μM of BrdU for 10 min and seeded onto poly-L-lysine-coated glass coverslips and fixed with 4% PFA for 20 min The coverslips were then treated with 2 N HCl for 15 min, neutralized in 0.1 M borate buffer (pH 8.5) and incubated for 90 min at 37 °C with rat anti-BrdU antibody (1:500; Abcam) in blocking buffer. Secondary antibodies (Supplementary Table 2) were incubated in blocking buffer for 1 h. Nuclei were counterstained with 1 mg ml^−1^ of 4,6-diamidino-2-phenylindole. For IGF2 staining, *Igf2* knockout SVZ sections were used to confirm antibody specificity. For SVZ whole mounts, we used protocols established previously^45^. Briefly, the lateral walls of the LV were dissected out and the resulting whole mounts were fixed for 1.5 h in 4% PFA and washed overnight at 4 °C in PB. Whole mounts were washed three times in PB containing 2% Triton X-100 for 15 min each, blocked for 2 h in 10% FBS and 2% Triton X-100 in PB, then incubated for 48 h at 4 °C with primary antibodies in the same blocking solution. After incubation with appropriate secondary antibodies, the stained walls were mounted with Fluorsave (Calbiochem) between two coverslips. 4,6-diamidino-2-phenylindole (1 mg ml^−1^, 5 min) was used for counterstaining. Samples were analysed using an Olympus FV100 confocal microscopes. For X-gal histochemistry, brains were fixed by immersion in 4% PFA in PBS at pH 7.4 with 2 mM MgSO4 and 5 mM EGTA for 30 min at 4 °C and processed for vibratome sectioning. Sections were incubated in PBS with 2 mM MgCl2, 5 mM K3Fe(CN)6, 5 mM K4Fe(CN)6, 0.01% sodium deoxycholate and 0.02% NP-40 and 1 mg ml^−1^ X-Gal for 24 h and counterstained with nuclear Fast Red.

### Gene expression studies

RNA was extracted with Trizol (Invitrogen), following the manufacturer’s guidelines. For quantitative PCR, 1 μg of total RNA was DNase-treated with RQ1 RNase-free DNase (Promega), following the manufacturer’s instructions. All complementary DNAs (cDNAs) were synthesized using random primers and SuperScript II RT reverse transcriptase (Invitrogen), following standard procedures. cDNA samples were diluted 1:10 and reactions were performed using Taqman Probes (Supplementary Table 3) in a Step One Plus cycler with Taqman Fast Advanced Master Mix (Applied Biosystems).

### Imprinting assay

*Igf2* imprinting assays were based on PCR amplification followed by direct sequencing to analyse parental-allele-specific gene expression. To determine the imprinting status of *Igf2* in the CP, we used the reciprocal F1 hybrid offspring of *Mus musculus domesticus* (strain C57BL/6, abbreviated BL6) and *Mus musculus castaneus* (strain CAST/EiJ, abbreviated Cast) subspecies, in which a single-nucleotide polymorphism (SNP) was identified between the two subspecies. The sequences of murine *Igf2* were obtained from GenBank (accession number NM_010514). PCR of the hybrid cDNA samples was performed using primers *Igf2SNP-F* (5′-CACGTCCCACACTAAGATCTCTC-3′) and *Igf2SNP-R* (5′-GGGGTGTCAATTGGGTTGT-3′) with the thermal cycler conditions: 94 °C 1 min, 60 °C 1 min, 72 **°**C 1 min and 30 cycles. The primer *Igf2SNP-R* was used for direct reverse PCR fragment sequencing. Genomic DNA sequence traces from BL6 and Cast placentae were used to identify the *Igf2* strain-specific polymorphism that was a guanine ‘G’ in BL6 mice and an adenine ‘A’ in Cast mice (Supplementary Fig. 2i) and located on exon 1.

### Cell cultures and co-culture experiments

Methods for NSC culture derived from SVZ and self-renewal assessment^40^. Briefly, brains were dissected out and the SVZ isolated in Earle’s balanced salt solution (Invitrogen). Tissues were transferred to Earle’s balanced salt solution containing 1.0 mg ml^−1^ papain (Worthington DBA), 0.2 mg ml^−1^ L-cystein (Sigma), 0.2 mg ml^−1^ EDTA (Sigma), incubated for 30 min at 37 °C and carefully triturated with a fire-polished Pasteur pipette to a single-cell suspension. Isolated cells were resuspended in DMEM/F12 medium containing 2 mM L-glutamine, 0.6% glucose, 9.6 g ml^−1^ putrescine, 6.3 ng ml^−1^ progesterone, 5.2 ng ml^−1^ sodium selenite, 0.025 mg ml^−1^ insulin, 0.1 mg ml^−1^ transferrin, 2 μg ml^−1^ heparin (sodium salt, grade II; Sigma) (control medium) and supplemented with 10 ng ml^−1^ of epidermal growth factor (human recombinant; Invitrogen) and 20 ng ml^−1^ of basic FGF (FGF2; human recombinant; Sigma) (complete medium). For each passage, spheres formed after 4–6 days *in vitro*. For self-renewal assays, neurospheres were treated with Accutase (0.5 mM; Sigma) for 10 min, mechanically dissociated to a single-cell suspension and replated in complete medium. SGZ-derived neurosphere cultures were obtained by dissecting out DG fragments and digesting in 0.025% trypsin and 0.265 mM EDTA (Invitrogen). A single-cell suspension was obtained by gentle trituration and diluted in complete medium. Neurospheres were allowed to develop for 6 days in a 95% air–5% CO_2_-humidified atmosphere at 37 °C. Some cultures were supplemented with recombinant Mouse IGF2 (50 ng ml^−1^; R&D Systems) or blocking anti-IGF2, anti-IGF1R and non-relevant antibodies (3 μg ml^−1^; Santa Cruz). Primary CP epithelial cells were cultured as previously described^42^. Briefly, five neonatal mice aged 7 days postpartum were killed by cervical dislocation, brains dissected out and CP transferred to a HBSS solution containing 2 mg ml^−1^ of Pronase. After 5 min incubation at 37 °C, choroid plexi were disaggregated by pipetting to generate a single-cell suspension, plated at 500 cells per millilitre and incubated overnight at 37 °C in 5% CO_2_/air to allow for cellular attachment. The medium containing unattached cells and debris was removed and cells were gently washed twice with PBS. Fresh new medium supplemented with 20 μM AraC was added to suppress fibroblast proliferation. The medium was changed every 48 h until the cells became confluent (7–10 days) at which point cultures were used for neurosphere co-culture assays. Cells were viable for several weeks with correct maintenance and without significant loss of marker gene expression or morphological features. Primary BECs were also obtained as previously described^41^. For co-culture experiments, the feeder CP epithelial or BECs’ media were replaced by neurosphere growth medium. After 2 h, a transwell insert (0.4 mm pore, 12 mm diameter; Millipore) was placed above, and dissociated NSCs were seeded in the upper compartment at a clonal density of 2.5 cells per microlitre. Neurosphere formation and diameter were analysed after 5 days incubation at 37 °C and 5% CO_2_.

### Enzyme-linked immunosorbent assay (ELISA)

The CP epithelial cells, BECs’ or NSCs media were collected after 4 days of conditioning and the levels of IGF2 were determined via ELISA following the manufacturer’s guidelines (IGF2 Mouse ELISA Kit, Abcam). Absorbances were measured using a VICTOR microplate reader (PerkinElmer) at 450 nm, and the amount of IGF2 was calculated from the standard curve in the detection limit range.

### Igf2 knockdown experiments and immunoblotting

Neurospheres freshly disaggregated were electroporated with lentiviral vectors carrying an Ig2-specific shRNA (TRC Clone number 0000071148, Sigma) or a non-target control (Mission SHC002, Sigma), using the Amaxa NSC Nucleofector kit and following the manufacturer instructions. Cells were starved for 6 h by removing growth factors and insulin 2 days after electroporation. Next, cells were treated with 20 ng ml^−1^ of IGF2 for 15 min. and washed with cold PBS. Cells were then lysed in RIPA buffer. Total protein concentration was determined using the BCA system (Pierce). Equal amounts (30 μg) of protein were loaded on polyacrylamide gels for SDS–polyacrylamide gel electrophoresis. Proteins were transferred to nitrocellulose membranes and immunoblots were carried out using primary antibodies (Supplementary Table 1) followed by incubation with appropriate secondary horseradish peroxidase-conjugated antibodies (Supplementary Table 2) and chemoluminiscent detection (Western Lightning, PerkinElmer). For immunoprecipitations, ∼10^6^ cells were split in two and either treated or not with 20 ng ml^−1^ IGF2 for 10 min, collected on ice in PBS supplemented with sodium orthovanadate and lysed in 25 mM Tris-HCl, pH 7.4, 150 mM NaCl, Triton X-100 1% and 1 mM EDTA supplemented with phosphatase and protease inhibitors. Lysates were incubated overnight with 3 μg of anti-IGFR1 antibody (1:1,000, Cell Signaling), and Dynabeads protein G (Invitrogen) were used to retrieve the immunoprecipitates. Beads were incubated with Laemmli buffer and separated from the samples using a magnet. Immunoprecipitates were resolved in 10% Criterion TGX Precast Gel (Bio-Rad) and transferred to nitrocellulose membranes (Protran Sigma). Membranes were blotted with primary anti-IGF2 and secondary peroxidase-conjugated antibodies (1:5,000; Bio-Rad) and reacted by chemiluminiscence. All antibodies were diluted in PBS containing 5% semi-skimmed milk and 0.025% Tween-20. Autoradiographic films were scanned and the bands were analysed by densitometry using Scion Image software. Uncropped western-blots are shown in Supplementary Fig. 9.

### Statistical analysis

All statistical tests were performed using the GraphPad Prism Software, version 4.00 for Windows ( http://www.graphpad.com). The significance of the differences between groups was evaluated in all experiments by a one-way analysis of variance followed by a Tukey’s *post hoc* test. When comparisons were performed with relative values (normalized values and percentages), data were normalized using an arcsen transformation. Treatment experiments were analysed by a paired *t*-test. Data are presented as the mean±s.e.m., and the number of experiments performed with independent cultures or animals (*n*) is indicated in the legends. The significance of the differences is provided: *P<0.05, **P<0.01, ***P<0.001.

## 

## Additional information

**How to cite this article:** Ferrón, S. R. *et al.* Differential genomic imprinting regulates paracrine and autocrine roles of IGF2 in mouse adult neurogenesis. *Nat. Commun.* 6:8265 doi: 10.1038/ncomms9265 (2015).

## Supplementary Material

Supplementary Information Supplementary Figures 1-10 Supplementary Tables 1-3
