# Supplementary material for: Differential genomic imprinting regulates paracrine and autocrine roles of IGF2 in mouse adult neurogenesis
Source: Nat Commun. 2015 Sep 15;6:8265. doi: 10.1038/ncomms9265 (PMC4579569; doi:10.1038/ncomms9265)
Supplement: Supplementary Information — Supplementary Figures 1-10 Supplementary Tables 1-3 [file ncomms9265-s1.pdf]

Supplementary Information

Differential genomic imprinting regulates paracrine and autocrine roles of IGF2 in mouse adult neurogenesis

Ferrón SR[1*], Radford EJ[2#], Domingo-Muelas A[1#], Kleine I[2], Ramme A[1], Gray D[2], Sandovici I[3,4], Constancia M[3,4,5], Ward A[6], Menheniott TR[7] and Ferguson-Smith AC[2,4*]

# contributed equally to this work

[1]Departamento de Biología Celular, Universidad de Valencia, Dr. Moliner, 50, Burjassot, 46100, Spain
[2]Department of Genetics, University of Cambridge, Downing St., Cambridge, CB2 3EH, UK
[3]Department of Obstetrics and Gynecology, University of Cambridge, Robinson Way, Cambridge, CB2 0SW, UK.
[4]Centre for Trophoblast Research, University of Cambridge, Downing St, Cambridge, CB2 3EG, UK
[5]NIHR Cambridge Biomedical Research Centre, Hills Road, Cambridge, CB2 0QQ, UK
[6]Department of Biology and Biochemistry, University of Bath, Claverton Down, Bath, BA2 7AY, UK.
[7]Murdoch Children's Research Institute, Royal Children Hospital, Parkville, Flemington Road, Victoria, 3052, Australia.

**To whom all correspondence should be addressed, at:*

Anne C. Ferguson-Smith
Department of Genetics
University of Cambridge
Downing Street
Cambridge CB2 3EG, UK
Tel: +44 1223 333 750
Fax: +44 1223 333 786
afsmith@gen.cam.ac.uk

Sacri R. Ferrón
Departamento de Biología Celular
Universidad de Valencia
46100 Burjassot, Spain
Tel: +34-963 543246
Fax: +34-963 543404
sacramento.rodriguez@uv.es

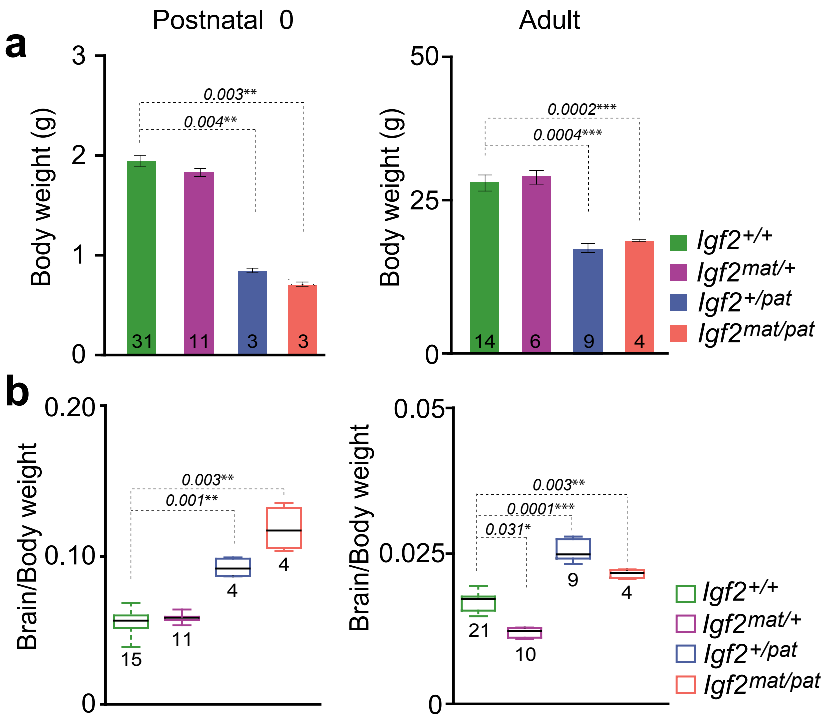

Supplementary Figure 1. Paternally expressed IGF2 controls post-natal and adult body weights. (**a**) Body weights in grams (g) of postnatal day 0 (left panel) and adult (2 months-old) (right panel) wild-type (*Igf2*$^{+/+}$), maternal transmission (*Igf2*$^{mat/+}$), paternal transmission (*Igf2*$^{+/pat}$) and homozygous knockout mice (*Igf2*$^{mat/pat}$). (**b**) Brain weights expressed relative to body weights of p0 (left panel) and adult (right panel) *Igf2*$^{+/+}$, *Igf2*$^{mat/+}$, *Igf2*$^{+/pat}$ and *Igf2*$^{mat/pat}$ mice. One way ANOVA and Tukey post-test. P-values and number of animals used per genotype are indicated. All error bars show s.e.m.

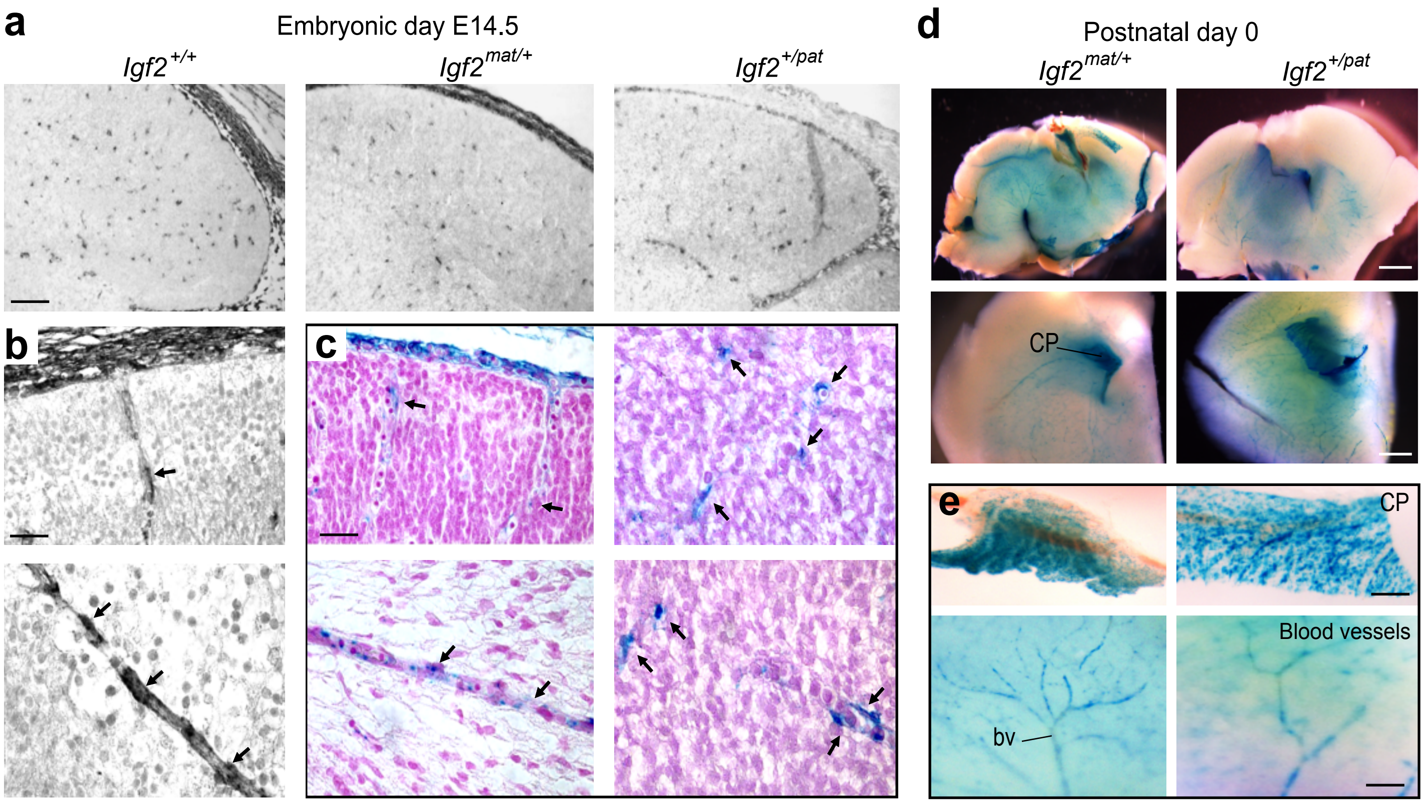

Supplementary Figure 2. ***Igf2 is biallelically expressed in embryonic and perinatal capillary endothelium, meninges and choroid plexus.*** **(a)** Histological sections were prepared from *Igf2*$^{+/+}$, maternal (*Igf2*$^{mat/+}$) and paternal (*Igf2*$^{+/pat}$) transmission E14.5 embryos and hybridised with an *Igf2* antisense RNA probe showing evidence of biallelic *Igf2* expression in endothelium as *Igf2* mRNA was detected when the embryo carried a maternal or paternal disruption of the endogenous gene. **(b)** High magnification views of *Igf2* mRNA expression of brain histological sections prepared from *Igf2*$^{+/+}$ E14.5 embryos, showing expression in meninges and capillary endothelium. **(c)** Detailed sagittal views of β-galactosidase staining (blue) in histological sections from *Igf2*$^{+/+}$, *Igf2*$^{+/pat}$ and *Igf2*$^{mat/+}$ E14.5 embryonic brains, showing biallelic *Igf2* expression in the capillary endothelium and meninges. Arrows indicate staining in individual capillaries. Sections were counterstained with nuclear fast red. **(d)** β-galactosidase staining (blue) in whole-mount preparations from maternal and paternal transmission *Igf2* heterozygote mice at postnatal day 0 (p0), showing biallelic *Igf2* expression in leptomeninges, **(e)** choroid plexus and blood vessels. CP: choroid plexus; bv: blood vessel. Scale bars in**:** a, 150 μm; b, c, 80 μm; d, 100 μm; e,50 μm.

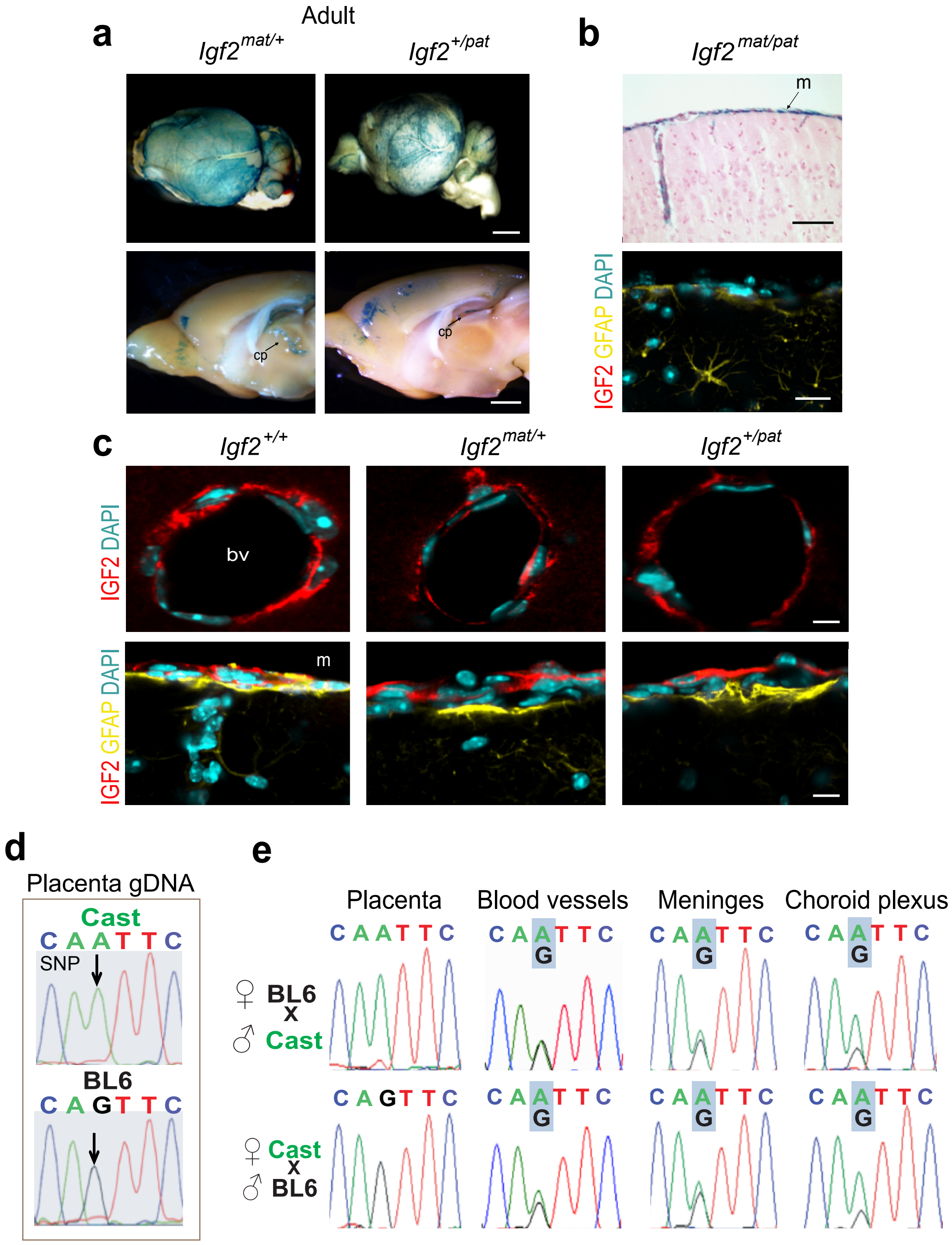

Supplementary Figure 3. *Igf2 is biallelically expressed in adult capillary endothelium, meninges and choroid plexus.* **(a)** Whole-mount staining (blue) for β-galactosidase activity in adult brains of *Igf2*$^{mat/+}$ and *Igf2*$^{+/pat}$ mutants (upper panels). Whole-mount staining for β-galactosidase (blue) in bisected hemispheres from *Igf2*$^{mat/+}$ and *Igf2*$^{+/pat}$ mice, showed significant activity of the enzyme in the choroid plexus independently of the parental origin of the mutation (lower panels). **(b)** β-galactosidase staining (blue) within the adult brain of *Igf2*$^{mat/pat}$ mice shows LacZ expression in the meninges in the brain (upper panel). Immunohistochemistry for IGF2 (red) and GFAP (yellow) in the meninges of *Igf2*$^{mat/pat}$ adult mice (lower panel). Homozygous knockout *Igf2*$^{mat/pat}$ mice were used for antibody control. **(c)** Immunohistochemistry for IGF2 (red) in coronal sections of vasculature within the brain in wild-type, *Igf2*$^{+/+}$, *Igf2*$^{mat/+}$ and *Igf2*$^{+/pat}$ mice (upper panels). Immunohistochemistry for IGF2 (red) and GFAP (yellow) in the adult meninges (lower panels). **(d)** Genomic DNA sequence traces showing the diagnostic strain-specific polymorphism (SNP) that was used for the detection of *Igf2* (A/G) imprinting using hybrid *Mus musculus domesticus* (abbreviated, BL6) and *Mus musculus castaneus* (abbreviated, Cast) mice. **(e)** *Igf2* allele specific expression in placenta at embryonic day 14 and adult blood vessels, meninges and choroid plexus derived from reciprocal F1 hybrid offspring from BL6 (G allele) and Cast (A allele). At least 4 tissue samples were sequenced. *Igf2* is known to be imprinted and paternally expressed in placenta. However, biallelic *Igf2* expression was observed in adult meninges, blood vessels and choroid plexus. DAPI was used for counterstaining. Scale bars in: f, 100 µm; g, upper panel: 100 µm, lower panel: 20 µm; h, 20 µm.

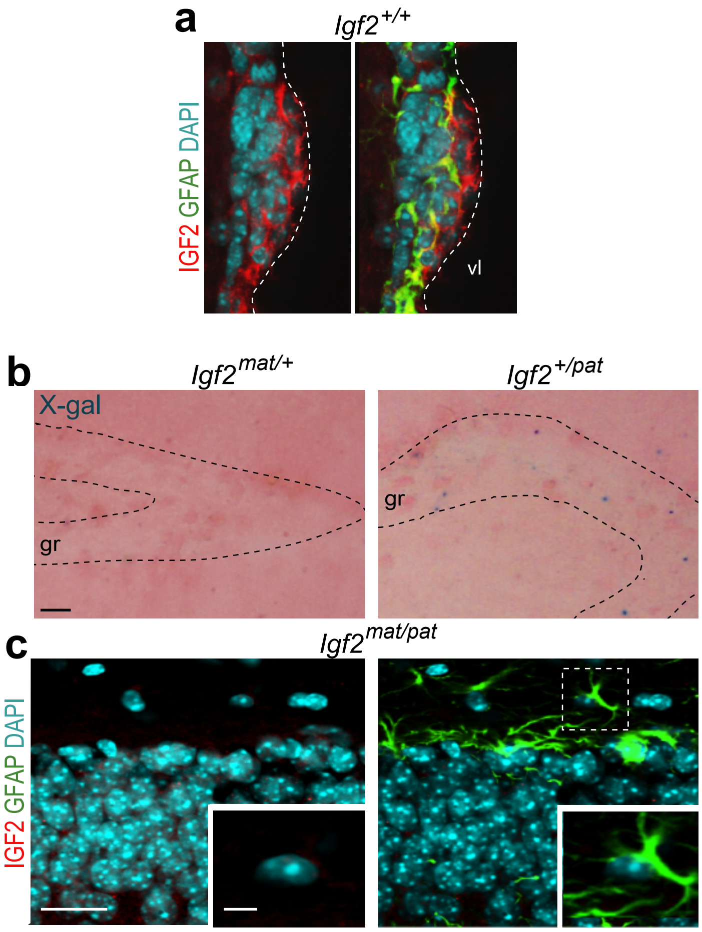

Supplementary Figure 4. *Igf2* is expressed from the paternal allele in the hippocampus. (a) Immunohistochemistry for IGF2 (red) and GFAP (green) in the SVZ of wild-type *Igf2*$^{+/+}$ mice. **(b)** β-galactosidase staining (blue) within the granular layer (gr) of the adult hippocampus of maternal transmission (*Igf2*$^{mat/+}$) and paternal transmission (*Igf2*$^{+/pat}$) heterozygote mice. **(c)** Immunohistochemistry for IGF2 (red) and GFAP (green) within the hippocampus of *Igf2*$^{mat/pat}$ mice showing that IGF2 is not expressed in knock-out mice. DAPI was used to counterstain nuclei. vl, ventricle lumen; gr, granular layer. Scale bars in: a, 20 μm; b, 70 μm; c, 30 μm (high magnification images: 7 μm).

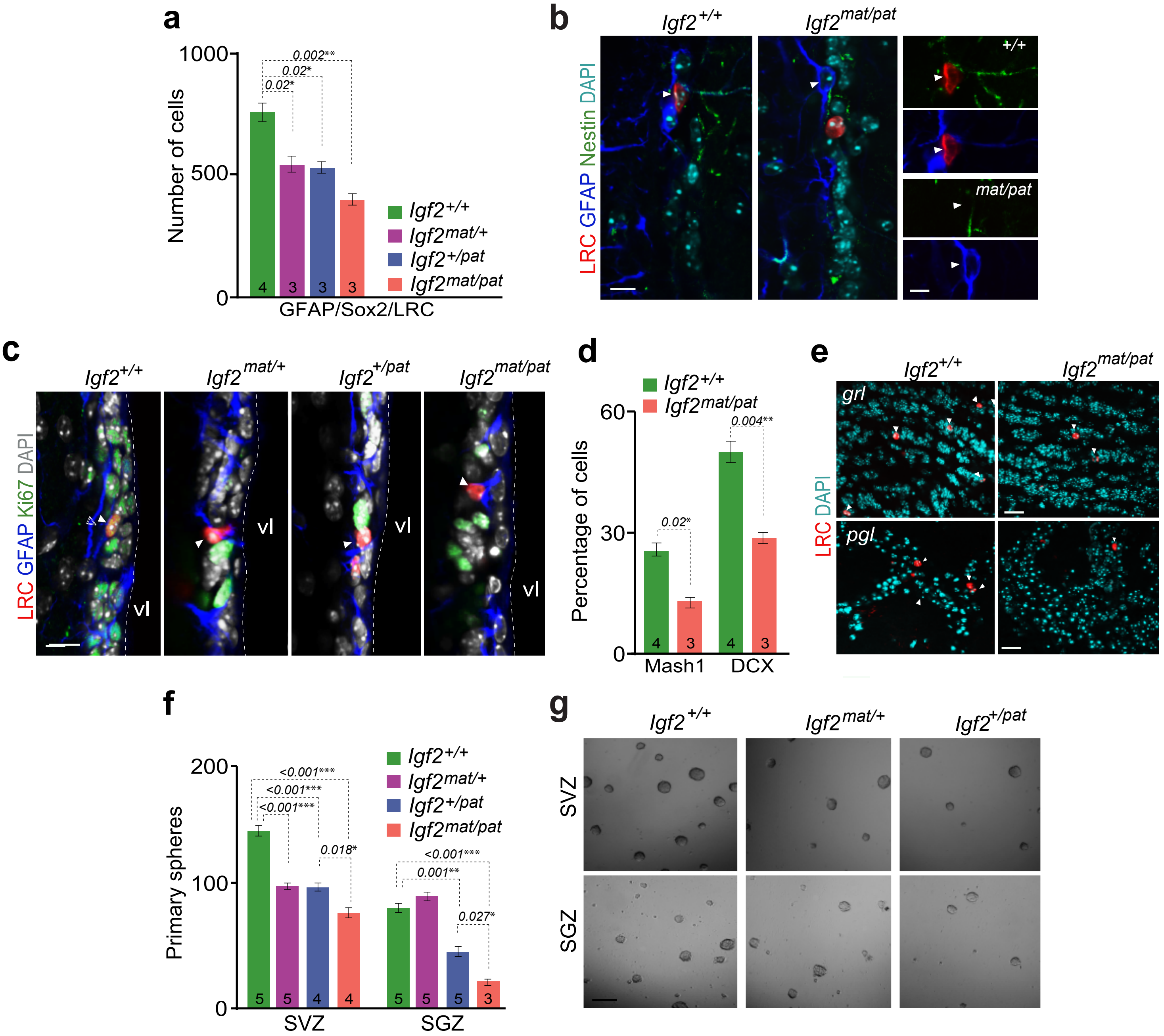

Supplementary Figure 5. Biallelic *Igf2* regulates olfactory bulb neurogenesis. (**a**) Quantification of the total number of CldU-Label retaining cells (LRC) that are GFAP+/SOX2+ in the SVZ of $Igf2^{+/+}$, $Igf2^{mat/+}$, $Igf2^{+/pat}$ and $Igf2^{mat/pat}$ mice. (**b**) Immunohistochemistry for CldU-LRC (red), GFAP (blue) and Nestin (green) within the SVZ of $Igf2^{+/+}$ and $Igf2^{mat/pat}$ mice (coronal plane). (**c**) Immunohistochemistry for CldU-LRC (red), GFAP (blue) and Ki67 (green) within the SVZ of $Igf2^{+/+}$, $Igf2^{mat/+}$, $Igf2^{+/pat}$ and $Igf2^{mat/pat}$ mice (coronal plane). (**d**) Quantification of the percentage of transit amplifying Mash1+ progenitor cells and DCX+ neuroblast population in the SVZ of wild-type and *Igf2* knock-out mice. (**e**) Immunohistochemistry for CldU-LRC (red) in the granular and periglomerular layers within the olfactory bulb in $Igf2^{+/+}$ and $Igf2^{mat/pat}$ mice (coronal plane). (**f**) Quantification of the number of primary neurospheres generated from the SVZ and the SGZ of $Igf2^{+/+}$, $Igf2^{mat/+}$, $Igf2^{+/pat}$ and $Igf2^{mat/pat}$ mice. (**g**) Representative phase contrast images of primary neurospheres isolated from the SVZ and SGZ of $Igf2^{+/+}$and *Igf2* heterozygote mice. DAPI was used to counterstain nuclei. vl, ventricle lumen; grl, granular layer; pgl, periglomerular layer. One way ANOVA and Tukey post-test. P-values and number of animals analyzed per genotype are indicated. All error bars show s.e.m. Scale bars in: b and c, 20μm; e, 30 μm; g, 60 μm.

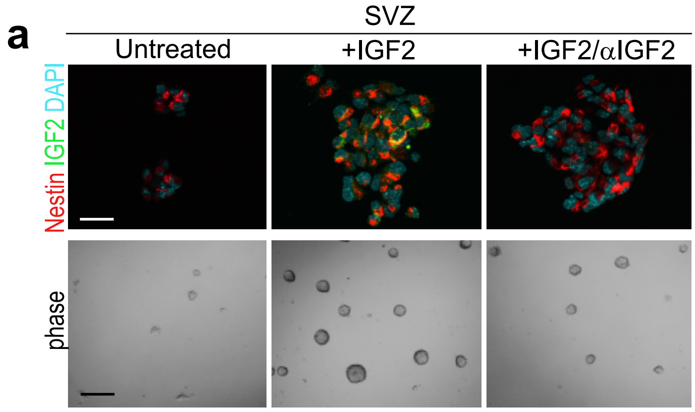

b

	Untreated	IGF2	IGF2/αIGF2	IGF2/NRA
Diameter	15.1 ± 4.3	43.4 ± 2.1**	26.7 ± 3.6	39.2 ± 3.2*
% Brdu	18.9 ± 2.7	50.6 ± 3.8**	27.7 ± 4.9	54.9 ± 3.4***
% Apoptosis	10.1 ± 3.0	11.4 ± 2.9	12.2 ± 5.1	10.4 ± 4.2

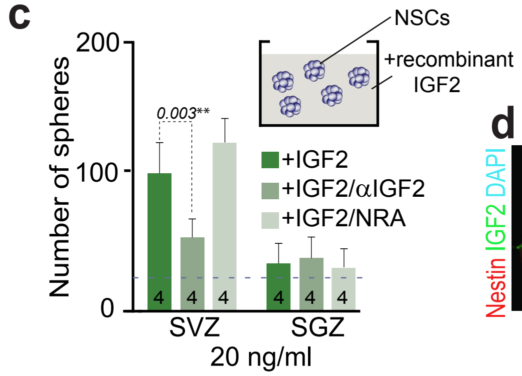

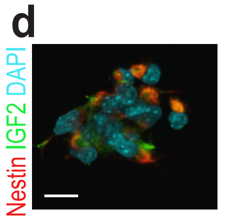

Supplementary Figure 6. SVZ-NSCs but not SGZ-NSCs respond to exogenous IGF2. (**a**) Immunocytochemistry for IGF2 (green) and Nestin (red) in neurospheres isolated from wild-type SVZ in the presence or absence of exogenously added IGF2 and after blocking with specific anti-IGF2 antibodies (upper panels). Representative phase contrast images of neurosphere cultures (lower panels). (**b**) Quantification of the mean sphere diameter, percentages of cells incorporating BrdU and apoptotic cells in SVZ neurospheres treated or untreated with exogenous IGF2. An increase in the proliferation of neurospheres after treatment with IGF2 was observed. (**c**) Schematic drawing of the IGF2 treatment experimental set up (upper panel). Quantification of the number of neurospheres formed after treatment with exogenous IGF2 (lower panel). An increase in the number of SVZ derived spheres was observed after treatment with the recombinant protein. Addition of anti-IGF2 antibodies abrogated this effect. No effect was observed in neurosphers from the SGZ of the hippocampus. Anti-CREB, a non-related antibody (NRA) was used as a negative control. (**d**) Immunocytochemistry for IGF2 (green) and Nestin (red) in neurospheres isolated from wild-type SGZ. All treatments were done in the absence of insulin and in combination with EGF and FGF in the media. Dashed line indicates untreated condition. Paired t-test. P-values and number of independent experiments are indicated. All error bars show s.e.m. Scale bars in: a, upper panels: 40 μm; lower panels: 60 μm; d, 40 μm.

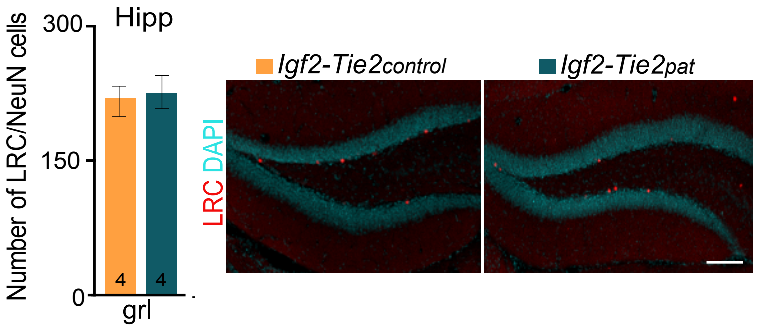

Supplementary Figure 7. IGF2 secreted by the endothelial niche does not regulate NSC self-renewal in the SGZ. (a) Quantification of the number of LRC in the granular layer (grl) in the hippocampus of *Igf2-Tie2control* and *Igf2-Tie2pat* mice (left panel). Immunohistochemistry for BrdU-LRC (red) is showing an overview of the dentate gyrus (DG) of the hippocampus of I*gf2-Tie2control* and *Igf2-Tie2pat mice* (right panel). One way ANOVA and Tukey post-test. All error bars show s.e.m. Number of animals analyzed per genotype is indicated. Scale bars:: 100 μM.

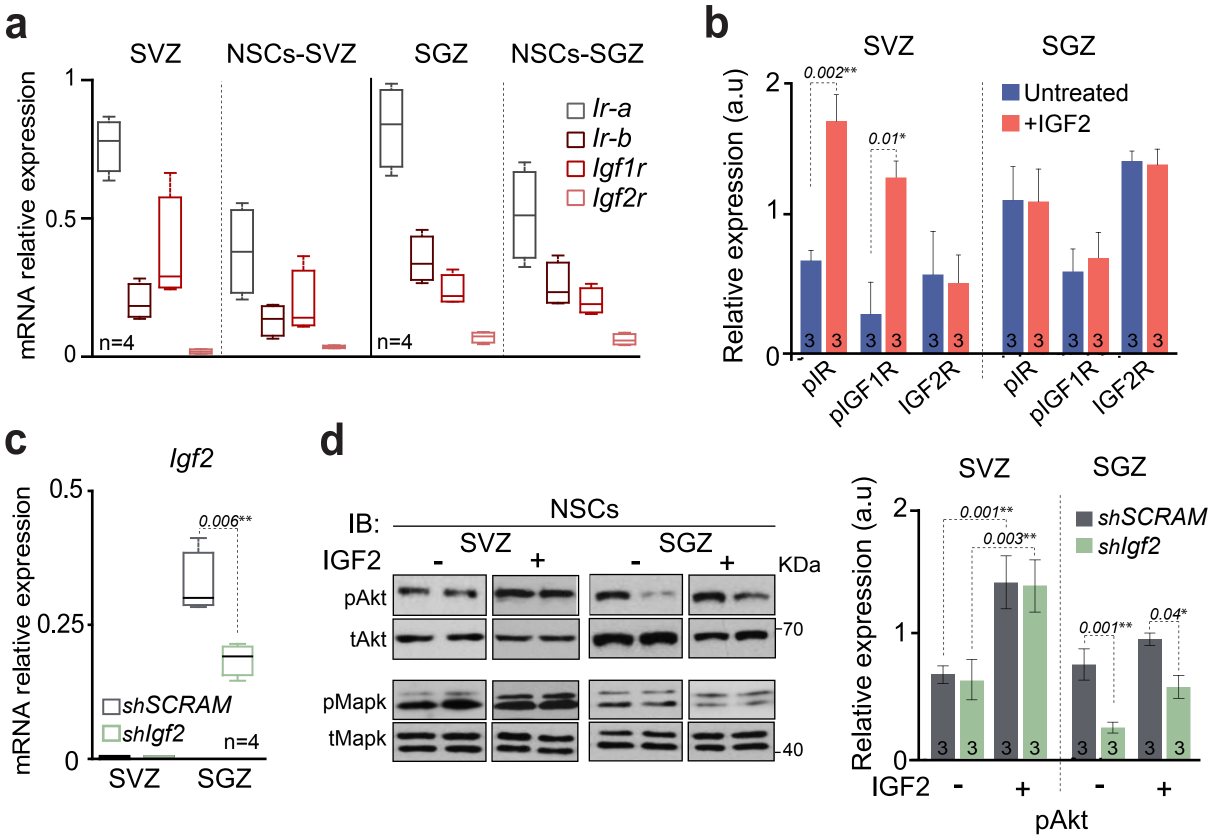

Supplementary Figure 8. IGF2 function involves IGF1R activation and pAkt induction. (a) PCR for insulin receptor isoforms a (*Ir-a*) and b (*Ir-b*), insulin growth factor like 1 (*Igf1r*) and 2 receptor (*Igf2r*) in NSCs derived from the SVZ and the SGZ, and in whole SVZ and hippocampus of adult wild-type mice. mRNA levels were normalized to β-*actin*. **(b)** Quantification of the levels of phospho-IR, phospho-IGF1R and IGF2R normalised to total IR, IGF1R or to GAPDH respectively in SVZ and SGZ-derived neurospheres after IGF2 treatment. **(c)** qPCR of *Igf2* expression in NSCs derived from the SVZ and SGZ that had been nucleofected with an *shRNAIgf2*. Downregulation of *Igf2* expression is observed in nucleofected NSCs. shSCRAMBLE was used as a control. **(d)** Detection of the levels of phospho-Akt (pAkt) and phospho-Mapk (pMapk) in SVZ and SGZ-derived neurospheres transfected with shRNA-*Igf2* and either with or without exogenous IGF2 (left panel). Quantification of the levels normalized to total Akt (Akt). Neurospheres nucleofected with a shSCRAMBLE (shSCRAM) were used as a negative control. Dashed line indicates number of spheres formed in the absence of IGF2.One way ANOVA and Tukey post-test. P-values and number of independent experiments or tissue samples are indicated. All error bars show s.e.m.

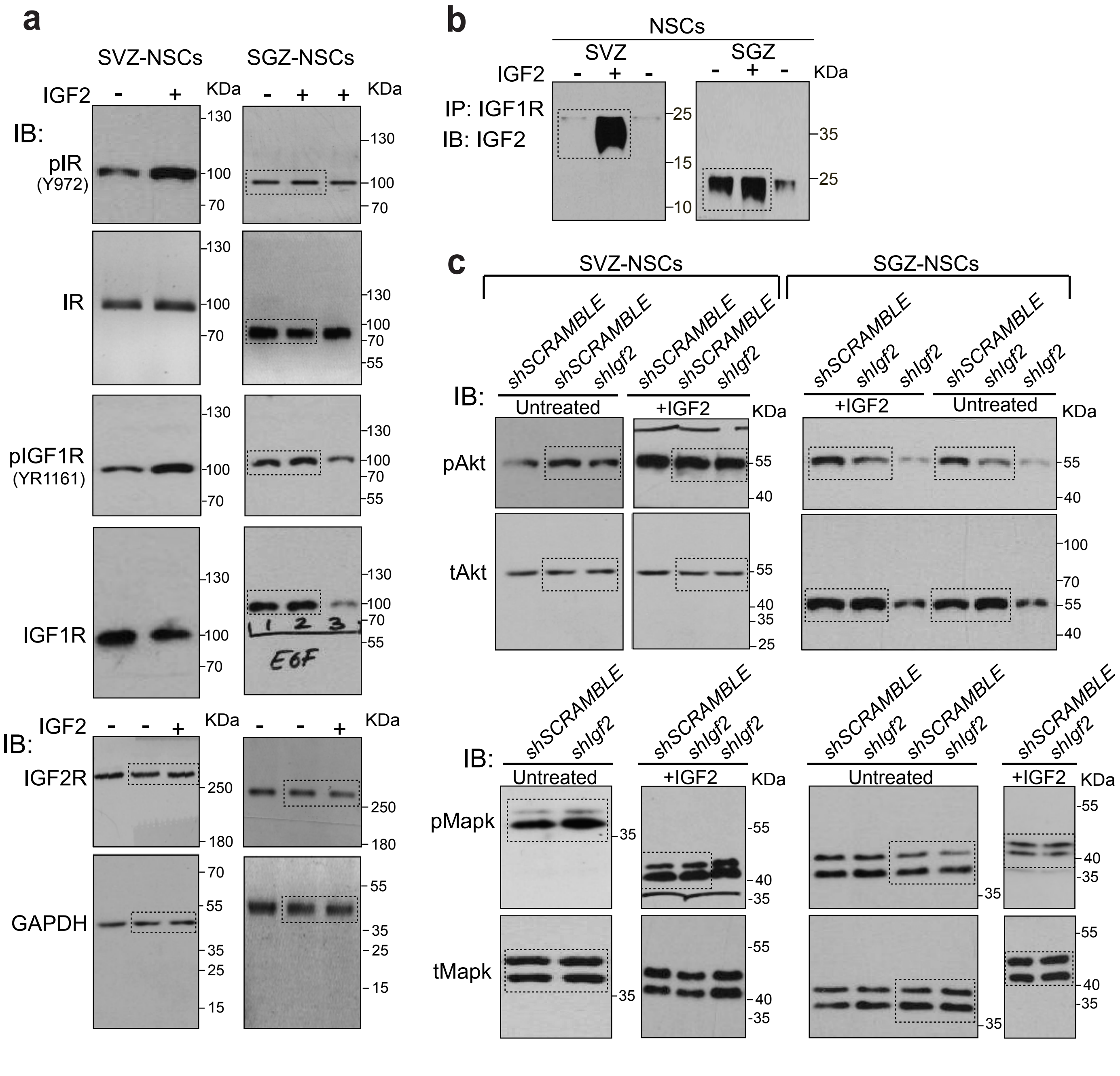

Supplementary Figure 9. Uncropped western blots. (a) Immunoblots (IB) in Figure 5e. **(b)** Immunoprecipitations (IP) followed by IB in Figure 5f. **(c)** Immnublots in Suplementary Figure 8d.

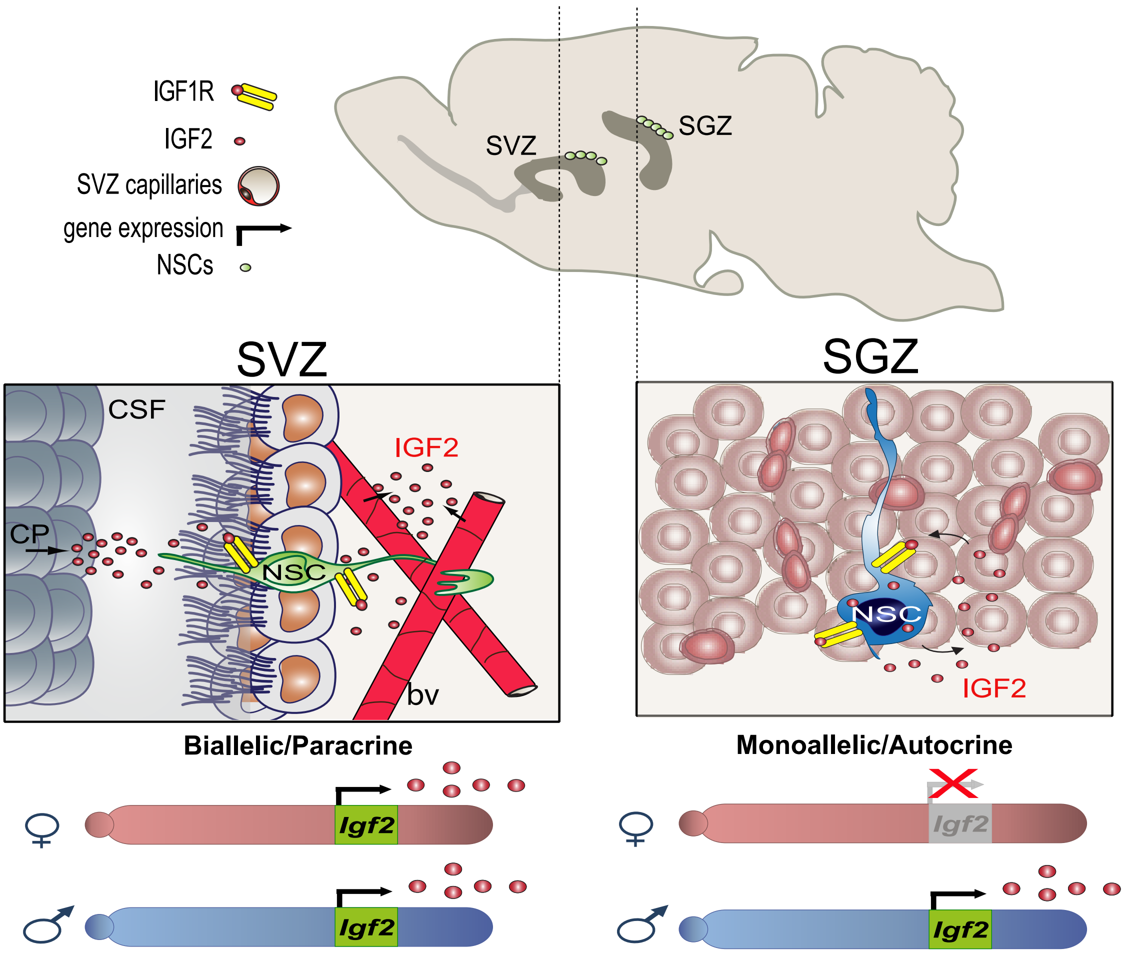

Supplementary Figure 10. Graphical abstract. In the SVZ, paracrine IGF2 is a cerebrospinal fluid (CSF) and endothelial-derived neurogenic factor requiring biallelic expression. In contrast, Igf2 is imprinted in the SGZ acting as an autocrine factor expressed in neural stem cells (NSCs) solely from the paternal allele.

Supplementary Table 1: List of primary antibodies.

Antibody	Type	Host	Dilution	Source	Catalog number	Application
**BrdU**	mAb	Rat	1:400	Abcam	ab6326	IHC
**CD-31**	mAb	Rat	1:100	BD Biosciences	550274	ICC/IHC
**DCX (C-18)**	pAb	Goat	1:300	Santa Cruz	sc-8066	IHC
**E-cadherin**	mAb	Mouse	1:100	BD Biosciences	610181	ICC
**GAPDH (14C10)**	mAb	Rabbit	1:5000	Cell Signaling	2118	WB
**GFAP**	pAb	Rabbit	1:500	Dako	Z0334	ICC
**GFAP**	pAb	Chicken	1:400	Millipore	AB5541	IHC
**IGF1R**	pAb	Rabbit	1:1000	Cell Signaling	3027	IHC/WB/B
**IGF2**	pAb	Rabbit	1:250	Abcam	ab9574	WB
**IGF2 (N-20)**	pAb	Goat	1:100	Santa Cruz	sc-1415	IHC/ICC
**IGF2R**	mAb	Mouse	1:500	Abcam	ab2733	IHC/WB
**IR (4B8)**	mAb	Rabbit	1:100	Cell Signaling	3025	IHC/WB
**MASH1**	mAb	Mouse	1:100	BD Biosciences	556604	IHC
**MKI67**	pAb	Rabbit	1:100	Abcam	ab15580	IHC
**NESTIN**	mAb	Mouse	1:3	Hybridoma Bank	Rat-401	IHC
**NeuN (A60)**	mAb	Mouse	1:250	Millipore	MAB377	IHC
**Pan-cytokeratin (H-240)**	pAb	Rabbit	1:200	Santa Cruz	sc-15367	ICC
**pY972IR**	pAb	Rabbit	1:500	Abcam	ab5678	WB
**pYR1161IGFIR**	pAb	Rabbit	1:300	Abcam	ab5681	WB
**SOX2**	pAb	Goat	1:100	R&D Systems	AF2018	IHC
**Transthyretin**	pAb	Rabbit	1:50	Dako	A000202	ICC
**β-catenin**	pAb	Rabbit	1:300	Cell Signaling	9587	IHC
**β-galactosidase**	mAb	Mouse	1:300	Promega	Z3781	IHC
**γ-tubulin (C-20)**	pAb	Goat	1:300	Santa Cruz	sc-7396	IHC
**Akt**	mAb	Mouse	1:1000	Cell Signaling	05-591	WB
**pAkt (Ser-473)**	mAb	Rabbit	1:1000	Cell Signaling	05-736	WB
**MapK**	pAb	Rabbit	1:2000	Cell Signaling	06-182	WB
**pMapK Erk1/2**	mAb	Rabbit	1:5000	Cell Signaling	04-797	WB

pAb: polyclonal antibody
mAb: monoclonal antibody
IHC: immunohistochemistry
ICC: immunocytochemistry
WB: western-blot
B: Blockade of Receptor-ligand Interaction

Supplementary Table 2. List of secondary antibodies.

Antibody	Source	Dilution
Alexa Fluor 488 Donkey anti-Mouse	Life Technologies	1:600
Alexa Fluor 488 Donkey anti-Rat	Life Technologies	1:600
Alexa Fluor 647 Donkey anti-Rabbit	Life Technologies	1:600
Alexa Fluor 647 Donkey anti-Chicken	Jackson ImmunoResearch	1:600
Cy3 Donkey anti-Goat	Jackson ImmunoResearch	1:800
Anti-Rabbit IgG (H + L)-HRP Conjugate	BioRad	1:5000
Anti-Mouse IgG (H + L)-HRP Conjugate	BioRad	1:3000

Supplementary Table 3. List of TaqMan probes

Gene	Amplicon (bp)	Applied Biosystems ID
Gapdh	107	Mm99999915_g1
Gapdh (VIC)	70	Mm03302249_g1
Igf1	77	Mm00439560_m1
Igf1r	106	Mm00802831_m1
Igf2	107	Mm00439564_m1
Igf2r	64	Mm00439576_m1
Ins2	99	Mm00731595_gh
Ins2r	57	Mm01211875_m1
Pecam1	71	Mm01242584_m1
